# *In vitro* Multi-Species Biofilms of Methicillin-Resistant *Staphylococcus aureus* and *Pseudomonas aeruginosa* and Their Host Interaction during *In vivo* Colonization of an Otitis Media Rat Model

**DOI:** 10.3389/fcimb.2017.00125

**Published:** 2017-04-18

**Authors:** Mukesh K. Yadav, Sung-Won Chae, Yoon Young Go, Gi Jung Im, Jae-Jun Song

**Affiliations:** ^1^Department of Otorhinolaryngology-Head and Neck Surgery, Korea University College of MedicineSeoul, South Korea; ^2^Institute for Medical Device Clinical Trials, Korea University College of MedicineSeoul, South Korea

**Keywords:** biofilms, planktonic, otitis media, methicillin resistant *Staphylococcus aureus*, *Pseudomonas aeruginosa*, poly-microbial, colonization

## Abstract

*Staphylococcus aureus* (SA) and *Pseudomonas aeruginosa* (PA) are known to cause biofilm-related infections. MRSA and PA have been frequently isolated from chronically infected wounds, cystic fibrosis, chronic suppurative otitis media (CSOM), and from indwelling medical devices, and these bacteria co-exist; however, their interaction with each-other or with the host is not well known. In this study, we investigated MRSA and PA multi-species biofilm communities *in vitro* and their interaction with the host during *in vivo* colonization using an OM rat-model. *In-vitro* biofilm formation and *in-vivo* colonization were studied using CV-microtiter plate assay and OM rat-model respectively. The biofilms were viewed under scanning electron microscope and bacteria were enumerated using cfu counts. The differential gene expressions of rat mucosa colonized with single or multi-species of MRSA or PA were studied using RNA-sequencing of total transcriptome. In multi-species *in-vitro* biofilms PA partially inhibited SA growth. However, no significant inhibition of MRSA was detected during *in-vivo* colonization of multi-species in rat bullae. A total of 1,797 genes were significantly (*p* < 0.05) differentially expressed in MRSA or PA or MRSA + PA colonized rat middle ear mucosa with respect to the control. The poly-microbial colonization of MRSA and PA induced the differential expression of a significant number of genes that are involved in immune response, inflammation, signaling, development, and defense; these were not expressed with single species colonization by either MRSA or PA. Genes involved in defense, immune response, inflammatory response, and developmental process were exclusively up-regulated, and genes that are involved in nervous system signaling, development and transmission, regulation of cell growth and development, anatomical and system development, and cell differentiation were down-regulated after multi-species inoculation. These results indicate that poly-microbial colonization induces a host response that is different from that induced by single species infection.

## Introduction

Globally, *Staphylococcus aureus* and *Pseudomonas aeruginosa* (PA) are two major opportunistic pathogens that cause community-acquired and nosocomial infections. *S. aureus* and PA are the most prevalent pathogens that colonize structurally abnormal airways such as those in cystic fibrosis (CF) and other chronic obstructive lung diseases (Lyczak et al., 2002; Hubert et al., 2013). In addition, they are frequently found together in chronic wound infections (Gjødsbøl et al., 2006; Fazli et al., 2009). *S. aureus* and PA cause biofilm-related infections, and methicillin-resistant *S. aureus* (MRSA) has emerged as a clinically relevant pathogen because of its resistance to antibiotics and its ability to form biofilms (Chopra et al., 2015). Bacteria within biofilms are difficult to eradicate because being encased in a polymer matrix decreases their susceptibility to antimicrobials and immune defenses; this inherent antimicrobial resistance provides added resistance to antimicrobial therapy and host defense. In addition, during infection, the bacteria that originate in biofilms disperse as planktonic cells, which results in spread to secondary sites and progression of the infection (Hall-Stoodley and Stoodley, 2009; Lister and Horswill, 2014). MRSA and PA have been detected in biofilm-related infections such as chronic suppurative otitis media (CSOM) and chronic middle ear infections (Jung et al., 2009; Kim et al., 2015). *S. aureus* and PA have been isolated from upper respiratory tract infections including several chronic diseases such as chronic otitis media, cholesteatoma, chronic adenoiditis, chronic sinusitis, post-operative trampansomay, and nasal polyposis (Post et al., 2004; Bendouah et al., 2006; Boase et al., 2013). In chronic rhinosinusitis (CRS) patients, multi-species biofilms have been associated with enhanced mucosal inflammation, more severe osteitis, higher incidence of recurrent infection (Li et al., 2011; Dong et al., 2014), and postoperative outcomes (Singhal et al., 2011), and post-surgery progression (Bendouah et al., 2006). Furthermore, *S. aureus* and PA have been isolated from multi-species biofilms that are frequently found on indwelling medical devices such as prostheses, stents, implants, catheters, and endotracheal tube (Percival et al., 2015). Although the effect of poly-microbial infections of *S. aureus* and PA has not been well studied, some studies suggested that such poly-microbial infections are more virulent than single species infections (Hendricks et al., 2001; Pastar et al., 2013). Voggu et al. (2006) reported that *in vitro* interactions between *S. aureus* and PA are competitive and result in PA eradicating *S. aureus* (Voggu et al., 2006). However, it has been reported that during polymicrobial colonization of PA and *S. aureus*, PA does not completely inhibit the colonization of *S. aureus*; rather, *S. aureus* employs numerous defense strategies for its survival in the same ecological niche and grows as a small-colony variant (SCV) (Biswas et al., 2009). As a result of the competitive interactions between *S. aureus* and PA, an altered colony morphology strains called small colony variants (SCVs) emerges. Those SCVs are more persistent and more antibiotic-resistant strains than normal *S. aureus* (Nair et al., 2014).

Multi-species biofilm infections have important implications for management because this association will modify the clinical course of the disease, affecting the selection of antimicrobial therapy and the anticipated response to treatment. However, despite the gravity of these multi-species infections, their pathogenesis remains largely unknown. Therefore, in this study, we investigated *S. aureus* and PA multi-species biofilm communities *in vitro*, and assessed their interaction with the host during *in vivo* colonization using an otitis media rat model.

## Materials and methods

### Ethics statement

All animal experiments were performed in accordance with the guidelines of the Animal Research Committee, Korea University College of Medicine, Seoul, South Korea. The animal experiment protocol was approved by the Institute Review Board of Korea University, Guro Hospital, Seoul, South Korea (Permit Number, KOREA 2016-0019).

### Bacterial strain and culture medium

Methicillin resistant *S. aureus* (MRSA) was purchased from the Culture Collection of Antimicrobial Resistant Microbes (CCARM 3903), Seoul, Korea. *P. aeruginosa* (ATCC 27853) was obtained from the American Type Culture Collection (Manassas, VA). The selective medium for MRSA was oxacillin resistance screening agar base (ORSAB) and *Pseudomonas*-specific medium was Pseudomonas agar base (PAB), and was purchased from Oxoid limited (Hampshire, UK). 2-n-heptyl-4-hydroxyquinoline-N-oxide (HQNO) was purchased from Santa Cruz Biotechnology (Dallas, Texas, USA).

### *In vitro* single species or multispecies biofilm growth of MRSA or PA

Single- or multi-species *in vitro* biofilm growth of MRSA and PA was established in 24-well (flat-bottom) polystyrene tissue culture plates (BD Falcon, Sparks, MD, USA) using a static model and a previously described procedure (Christensen et al., 1985; Yadav et al., 2015b). The biofilm biomass was quantified using a crystal violet (CV) microtiter plate assay, and the bacterial loads within biofilms were enumerated by colony forming unit (CFU) counts. MRSA or PA cell suspensions (1 × 10^7^), individually or in combination in TSB media, were inoculated (1 mL) in 24-well polystyrene plates. The plates were incubated at 37°C for 24 h. After incubation, medium was discarded, and plates were gently washed with 1 mL sterile water. Thereafter, plates were air-dried and stained with 200 μL CV (0.1%) for 15 min. Excess stain was decanted, and plates were washed three times with sterile distilled water. The biofilm was dissolved in 1 mL (95%) ethanol and the optical density (OD) at 570 nm was measured in an automatic spectrophotometer. All experiments were performed in triplicate and the average was calculated. The experiments were repeated three times.

Alternatively, MRSA, PA, or combinations of both species were grown in TSB medium under the same conditions. CFUs were counted to quantify the number of viable cells growing in the biofilms. Biofilms were dissolved with sonication at 50 W for 10 s, serially diluted, and plated on selective medium, specifically ORSAB or PAB with CN supplement, to determine CFU-values.

To characterize the biofilm matrix, 24-h pre-established biofilms of MRSA or PA were treated with 10 mM sodium metaperiodate (Sigma, St. Louis, MO, USA), 100 μg/mL DNase I (Roche, Mannheim, Germany), 100 μg/mL alginate lyase (sigma), and 100 μg/mL proteinase K (sigma) by procedure previously described (Gutiérrez et al., 2014). The control biofilms were treated with respective buffer. The biofilms biomass was quantified as described above.

### Biofilm growth of SCVs of MRSA

*S. aureus* forms SCVs in the presence of HQNO produced by PA (Mitchell et al., 2010). To evaluate biofilm formation capability of SCVs of MRSA (3903), biofilms were grown with (20 μg/mL) HQNO, whereas the control biofilm was grown with DMSO supplementation. Biofilm biomass was quantified using a CV-microtiter plate assay as described above. The morphological variations in SCV biofilms grown with HQNO were examined using a scanning electron microscope (SEM).

### *In vivo* single species or multispecies colonization of MRSA or PA in the rat middle ear

*In vivo* single species or multispecies colonization by MRSA or PA was assessed using a previously described otitis media (OM) model (Yadav et al., 2012, 2014, 2015a). Forty pathogen-free Sprague-Dawley rats weighing 150–200 g were obtained from Koatech (Pyeongtaek, South Korea). Before the experiment, all animals were examined for abnormalities in the middle ear and were housed in an infection-free zone for 2 weeks. The rats were divided into four experimental groups. Group 1 included rats inoculated with MRSA only (*n* = 11); Group 2 was rats inoculated with PA only (*n* = 11); Group 3 included rats inoculated with a mixed culture of MRSA + PA (*n* = 11); and Group 4 was rats inoculated with media only (vehicle control; *n* = 7). The bacterial cell suspensions were prepared in TSB medium. Cell suspension (50 μL) containing 1 × 10^7^ CFU of either single species MRSA, PA, or mixed culture of the *two* species was injected into the middle ear cavity through the tympanic membrane of the right ear using a tuberculin syringe and a 27-gauge needle. The animals were euthanized using a 1:1 combination of nitrous oxide and oxygen. One week after inoculation, the rats were sacrificed using carbon dioxide inhalation, and the bulla was aseptically acquired. The tympanic membrane was removed, and the ears were irrigated to remove planktonic bacteria. The bullae from each group were dissected and cleaned, and the middle ear was visualized and photographed. The bullae were homogenized, serially diluted, and plated on selective plates to obtain CFU counts. For SEM analysis, the bullae were preserved in SEM solution.

### Visualization of *in-vitro* biofilms and *in-vivo* colonization of MRSA or PA single species or multi-species using SEM

MRSA or PA single species or multispecies *in vitro* biofilms grown in 24-well tissue culture plates for 24 h were analyzed by SEM. After a 24-h incubation, the medium with planktonic cells were discarded, and the plates were gently washed twice with sterile PBS. Samples were pre-fixed by immersion in 2% glutaraldehyde and 2.5% paraformaldehyde solution, and post-fixed for 2 h in 1% osmic acid dissolved in PBS. Samples were treated with a graded series of ethanol and *t*-butyl alcohol. The samples were freeze-dried in a freeze dryer (ES-2030 Hitachi, Tokyo, Japan), followed by platinum coating using an IB-5 ion coater (Eiko, Kanagawa, Japan), and specimens were visualized using a S-4700 field emission scanning electron microscope (Hitachi).

Similarly, for SEM analysis of *in vivo* colonization by MRSA or PA, the rat bullae from representative groups were dissected and cleaned of unwanted tissue, and the middle ear was carefully opened. The bullae were preserved in glutaraldehyde and paraformaldehyde solution. The rest of the protocol (pre-fix, post-fix, dehydration, etc.) was the same as that described previously for *in vitro* biofilms.

### Total RNA extraction and transcriptome sequencing using rat mucosa colonized with single or multi-species of MRSA or PA

Rats from representative groups were sacrificed as described above, and the bullae were acquired. Bullae were immediately dissected, and the middle ear was exposed and preserved in RNAlater (Qiagen, Hilden, Germany). The mucosal membrane of the bullae preserved in RNAlater solution was carefully scraped and preserved in fresh RNAlater solution until RNA extraction. Total RNA was isolated using a Qiagen RNeasy kit (Qiagen, Hilden, Germany) in accordance with the manufacturer's instructions. RNA was quantified using a nano-drop, and the RNA quality was assessed by analysis of rRNA band integrity using an Agilent RNA 6000 Nano kit (Agilent Technologies, Palo Alto, CA, USA). Ahead of cDNA library construction, 2 μg of total RNA and magnetic beads with Oligo (dT) were used to enrich poly(A)-containing mRNA. Subsequently, the purified mRNA was disrupted into short fragments, and double-stranded cDNA was immediately synthesized. The cDNA was subjected to end-repair and poly(A) addition, and was connected with sequencing adapters using the TruSeq RNA sample prep kit (Illumina, CA, USA). Suitable fragments that were automatically purified using a BluePippin 2% agarose gel cassette (Sage Science, MA, USA) were selected as templates for PCR amplification. The final library sizes and qualities were evaluated electrophoretically using an Agilent high sensitivity DNA kit (Agilent Technologies, CA, USA) and the fragment was determined to be between 350 and 450 bp. Subsequently, the library was sequenced using an Illumina HiSeq2500 sequencer (Illumina, CA, USA).

Low quality reads were filtered according to the following criteria; reads containing more than 10% of skipped bases (marked as “N”s), reads containing more than 40% of bases with quality scores <20, and reads with average quality scores <20. The entire filtering process was performed using the in-house scripts. Filtered reads were mapped to the human reference genome (Ensembl release 72) (Flicek et al., 2013) using aligner STAR v.2.3.0e (Dobin et al., 2013).

Gene expression levels were measured with Cufflinks v2.1.1 (Trapnell et al., 2010) using the gene annotation database of Ensembl release 72. The non-coding gene region was removed with the mask option. To improve the accuracy of measurement, multi-read-correction and fragbias-correct options were applied, whereas all other options were set to default values. For differential expression analysis, gene level count data were generated using the HT Seq-count v0.5.4p3 (Anders et al., 2015) tool with the option “-m intersection-nonempty” and -r option, considering paired-end sequences.

Based on the calculated read count data, differentially expressed genes (DEG) were identified using the R package TCC (Sun et al., 2013). The TCC package applies robust normalization strategies to compare tag count data. Normalization factors were calculated using the iterative DEGES/edgeR method. Q-values were calculated based on the *p*-value using the p.adjust function of the R package with default parameter settings. DEGs were identified based on a q-value threshold < 0.05. Gene ontology (GO) and KEGG pathway analysis of the DEGs were performed using STRING version 10.

### Quantification of gene expression using real-time RT-PCR

RNA-sequencing gene expression results were confirmed by real-time RT-PCR. Eighteen DEGs, and one house-keeping gene, glyceraldehyde-3-phosphate dehydrogenase (GAPDH), were analyzed by real-time RT-PCR. The gene sequences were downloaded from the NCBI gene bank, and primers were designed using primer express software (Table 1). cDNA synthesis was performed using a PrimeScript first strand cDNA synthesis kit according to the manufacturer's instructions (TaKaRa Bio Inc., Kusatsu, Japan). The amplification reaction was performed in an ABI Prism 7300 real-time PCR system (Applied Biosystems, Foster, CA, USA) in a total volume of 20 μL. The reaction mixture consisted of 10 μL of 2 × SYBR Green PCR Master Mix (Roche Applied Science, Indianapolis, IN, USA), 2.5 pmol of each forward and reverse primer, and 4 μL cDNA. The PCR reaction mixture was incubated as follows: initial denaturation at 95°C for 10 min, followed by 45 cycles of DNA denaturation at 95°C for 15 s, primer annealing and extension at 56°C for 1 min, and a final extension step at 72°C for 5 min. A control reaction without reverse transcriptase was included in each real-time RT-PCR experiment to check for cDNA contamination with genomic DNA. The relative quantification of gene expression in rat mucosa inoculated with *S. aureus*, PA, or mixed culture, with respect to the type of media, was performed using the 2^−ΔΔCT^ method (Livak and Schmittgen, 2001), and the gene expression values were normalized to those of the housekeeping gene, GAPDH.

**Table 1:** **List of primers used in this study**.

**Serial number**	**Gene name**	**Sequence**	**Base pairs**	**Amplicon size**
1	*KERA*	F: CCG TCG AGG GGT TTT GAT GT	20	246
		R: CAT CGG GAT TGG TGG CTT GA	20	
2	*GPD1*	F: AAC AGG TGC TGA CAT CCT GG	20	254
		R: AGC CAA TGG TCG TCT CAC AG	20	
3	*ACTA1*	F: CTC TTG TGT GTG ACA ACG GC	20	124
		R: CCC ATA CCG ACC ATG ACA CC	20	
4	*EEF1A2*	F: TCG GAT CCT CGT TAC GCC G	19	195
		R: GCC GGC GGT TTT ATC TCT CT	20	
5	*HRC*	F: CTT CCA GGA GCC ATG GTT GT	20	138
		R: CCA GGG ATA CCT GCG TTG TT	20	
6	*FOXD1*	F: GGT ACT CTG CAC CAA GGG AC	20	130
		R: CCC ATC CGT AGA AAG GAG CC	20	
7	*MYH1*	F: AGT TGC ATC CCT AAA GGC AGA	21	148
		R: GGC TTG TTC TGA GCC TCG AT	20	
8	*SYNPO2L*	F: GGG TAC CAG CAC CTC AAC TT	20	233
		R: TAA GAG CTG GTC CCT CTC CC	20	
9	*TNMD*	F: GTC CCA CAA GTG AAG GTG GA	20	148
		R: TGC CTC GAC GGC AGT AAA TA	20	
10	*MYOM2*	F: GGT ACT CCT CAT CTT TCT GGG AA	23	134
		R: TCG ATG CAT ATC GGT CCA GG	20	
11	*RPS9*	F: CGT TTC TCT TTG TCA CGG GC	20	192
		R: CCT CCA CAC CTC ACG TTT GT	20	
12	*MMP12*	F: CTC CCA TGA ACG AGA GCG AA	20	170
		R: GGT GTC CAG TTG CCC AGT TA	20	
13	*ITGB2*	F: TTG TCA ACA CCC ATC CCG AG	20	215
		R: AAT TTC CTC CGG ACA GGC AG	20	
14	*CST7*	F: GCA TAC ACC TCA GAT TTT TGT TCC A	25	235
		R:TAG TTC GGC CGA TTT CCA CC	20	
15	*SCTR*	F: GTC ATT CGA GGG CCT GTG AT	20	197
		R: GGG GAG AAG GCG AAG ACA AT	20	
16	*APOC1*	F: CAT AGT GGT GGG AGG TGG TG	20	103
		R: AGG AAG TGC GAT GAA GAG CC	20	
17	*CD69*	F: GCGATATGCTGGTGGACTGA	20	111
		R: GACCCTGTCACGTTGAACCA	20	
18	*CCNO*	F: CCAGTCGTTGCAGCCCATTA	20	261
		R: ACCTCTCGGCAAGTCAAAGG	20	

### Statistical analysis

Values were calculated as the mean of individual experiments that were performed in triplicate, and compared to those of the control groups. Differences between mean values were assessed using a Student's *t*-test. Statistical significance was set at *p* < 0.05.

## Results

### *In vitro* single species or multispecies biofilm growth of MRSA or PA

**Figure 1:**
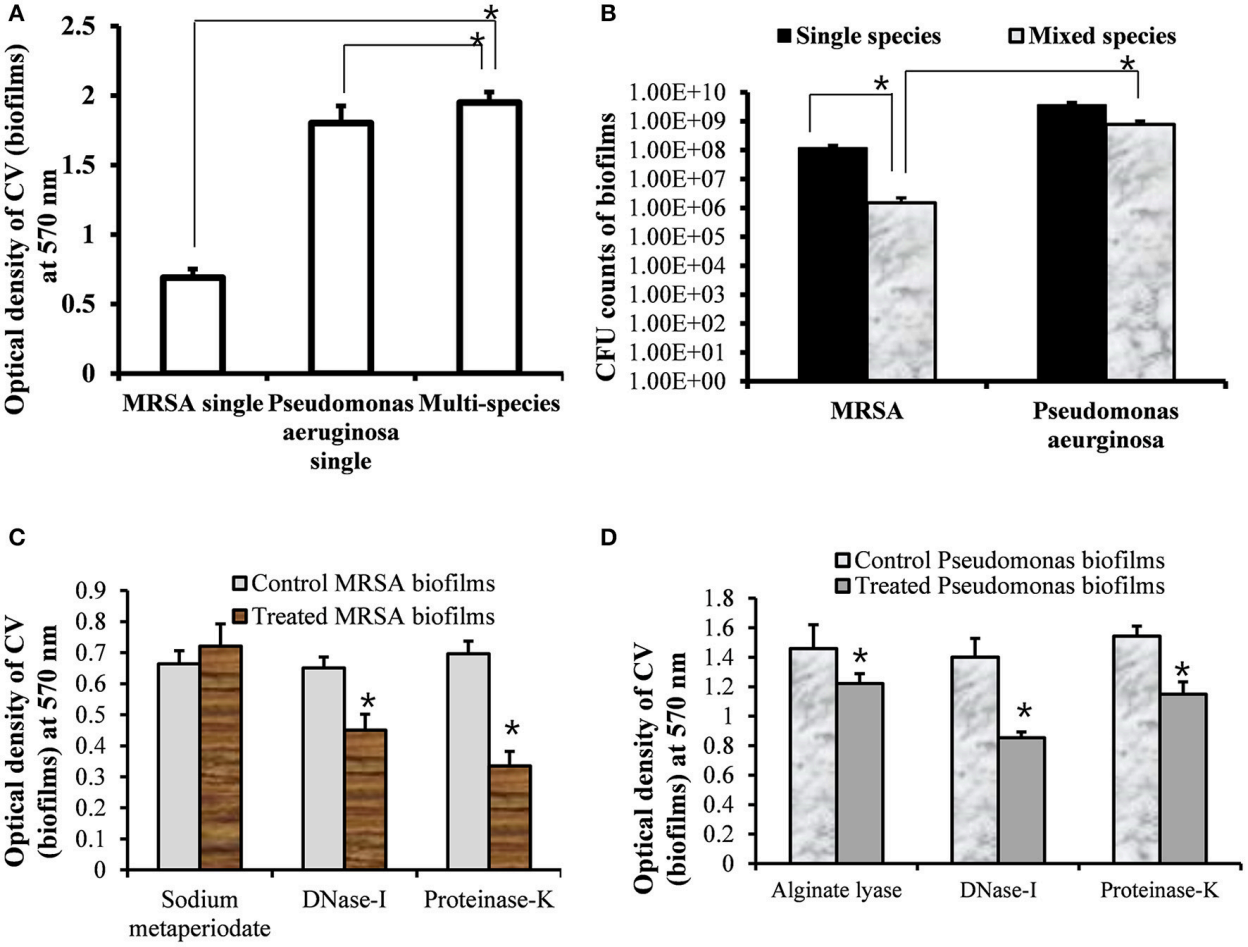
**Quantification of single- and multi-species *in vitro* biofilms of methicillin-resistant *Staphylococcus aureus* (MRSA) and *Pseudomonas aeruginosa* (PA). (A)** Quantification of single-species and multi-species biofilms of MRSA or PA using crystal violet microtiter plate assays. **(B)** Colony forming unit (CFU) counts of bacteria within single- and multi-species biofilms of MRSA or PA. **(C)** Eradication of pre-established MRSA biofilms with sodium metaperiodate (SM), DNase I and proteinase K with respect to control (untreated). **(D)** Eradication of PA pre-established biofilms with alginate lyase, DNase I, and proteinase K. The error bars represent the standard deviation from the mean value. The results were compared using a Student's *t*-test (^*^p < 0.05).

To assess the growth of MRSA and PA in single and multi-species biofilms, *in vitro* single and multi-species biofilms were grown on polystyrene plates that allowed for bacterial attachment and biofilm formation. The biofilms were quantified using a CV-microtiter plate assay after removing planktonic cells. The CV-microtiter plate assay detected significantly (*p* < 0.05) increased biofilm biomass in multi-species biofilms of MRSA and PA, compared to single species biofilms of either MRSA or PA (Figure 1A). The OD_570_-values of single species biofilms of MRSA and PA were 0.7 and 1.8, respectively; however, that of the multi-species biofilm was 2.0. These results indicated that total biomass of multi-species biofilms were higher than that of single species biofilms, which might be because of the accumulation of extracellular polysaccharide (EPS) and cells from both species. Thus, both species contributed to increased biofilm biomass.

To further evaluate the viable bacteria in single or multi-species biofilms, CFUs were enumerated. The CFU counts showed significantly higher numbers of bacteria in single species biofilms than in multi-species biofilms of either MRSA or PA (Figure 1B). In single species biofilms, the CFU counts for MRSA and PA were 1 × 10^8^ and 4 × 10^9^, respectively. In multi-species biofilms, PA inhibited the growth of MRSA. The CFU counts of MRSA and PA in multi-species biofilms were 1 × 10^6^ and 8 × 10^8^, respectively. The CFU counts for PA were significantly (*p* < 0.05, approximately 2.6 log values) higher than those of MRSA. These results indicated that PA and MRSA could exist together in multi-species *in vitro* biofilms. However, the decreased CFU counts for MRSA indicated that PA partially inhibited the growth of MRSA in multi-species biofilms.

The treatment of pre-established biofilm of MRSA with DNase I and Proteinase K significantly (*p* < 0.05) decreased biofilm biomass in compare to control biofilms (Figure 1C). However, no effect of sodium metaperiodate was detected. These results indicate that MRSA biofilms contains e-DNA and proteins, and the MRSA produce *ica*- independent biofilms. Previous studies reported no effect of sodium metaperiodate on MRSA biofilms (Fitzpatrick et al., 2005). Treatment of PA biofilms with DNase I and proteinase K significantly (*p* < 0.05) reduced biofilms biomass. However, very low biofilm eradication activity of alginate lyase was detected on pre-established biofilms of PA (Figure 1D). PA is known to produces at least three types of exopolysaccharides. The mucoid PA strains predominantly produce alginate exopolysaccharide (Hentzer et al., 2001), and non-mucoid strains produce the Pel and Psl polysaccharides for biofilm formation (Wozniak et al., 2003). ATCC 27853 is a standard non-mucoid strain (Li et al., 2001). These results indicate that PA (ATCC 27853) biofilms contains DNA and proteins along with polysaccharides primarily the Pel and Psl.

**Figure 2:**
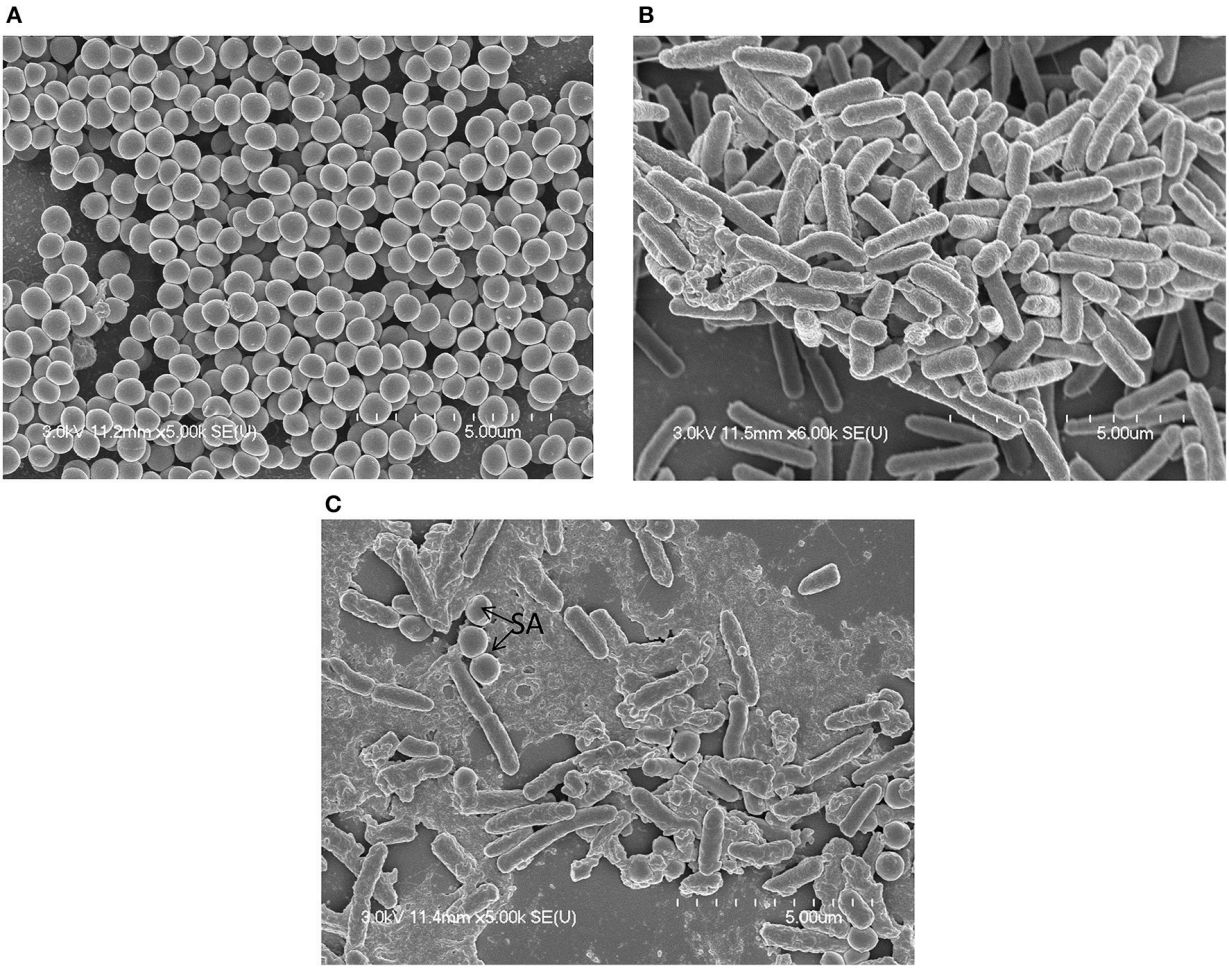
**Scanning electron microscope (SEM) images of *in vitro* biofilms of single or multi-species methicillin-resistant *Staphylococcus aureus* (MRSA) and *Pseudomonas aeruginosa* (PA). (A)** Representative SEM image of MRSA single species biofilms. **(B)** Representative SEM images of PA single species biofilms. **(C)** Representative SEM images of multi-species biofilms of MRSA and PA.

MRSA or PA single-species or multi-species *in vitro* biofilms were analyzed using SEM. SEM analysis revealed that single-species biofilms were compact, and cells were connected to the bottom of the plate and to each other (Figures 2A,B). The multi-species biofilms were also compact, and the bacteria were interconnected to each other and to the surface. In multi-species biofilms, the biofilm debris or may be EPS was visible that were not visible in single species biofilms of either MRSA or PA (Figure 2C). As expected in multi-species biofilms, fewer *S. aureus* bacteria were visible than PA, indicating that PA partially inhibited MRSA growth in the biofilm state.

### SCVs of MRSA enhance biofilm formation

**Figure 3:**
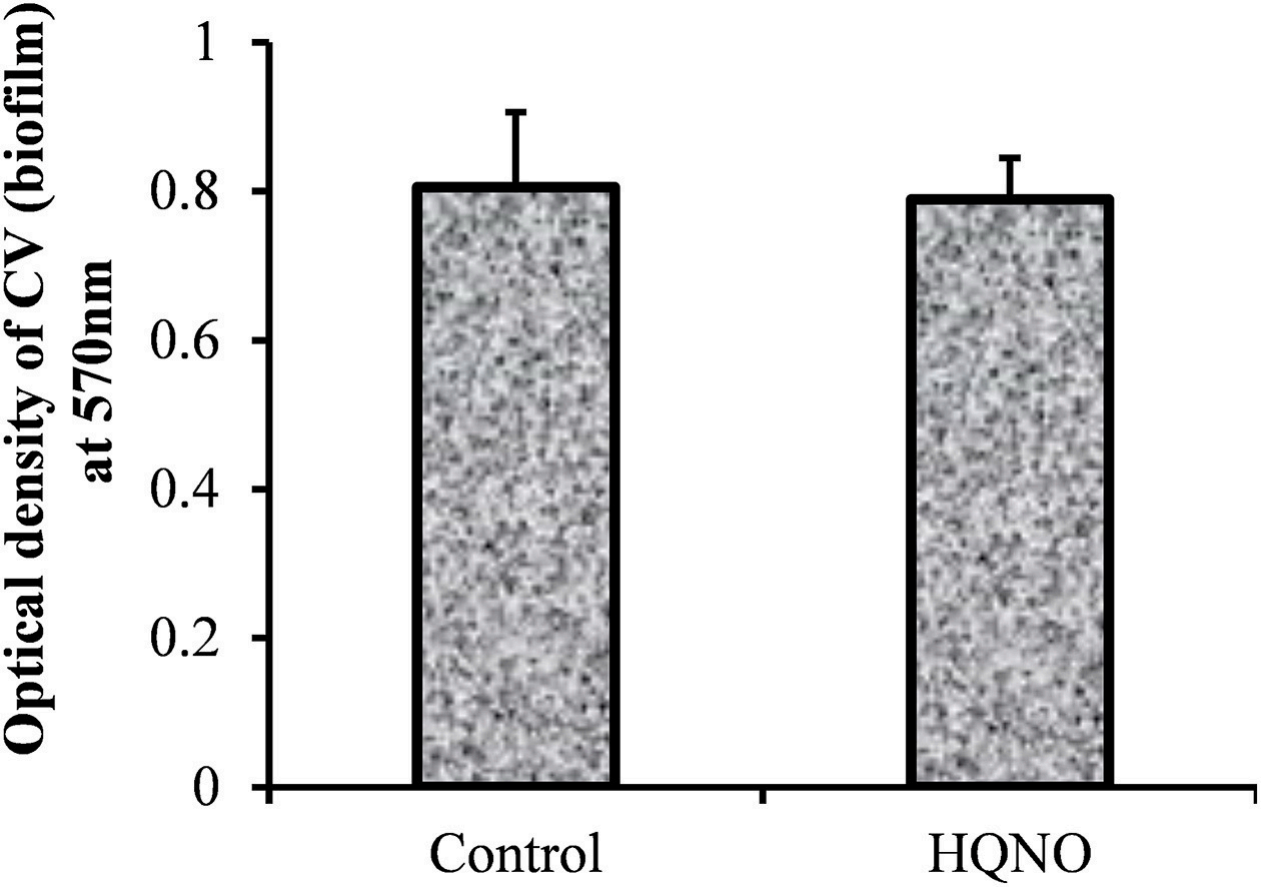
***In vitro*** **biofilm growth of methicillin-resistant *Staphylococcus aureus* (MRSA) small colony variant**. *In vitro* biofilm growth of MRSA supplemented with HQNO (20 μg/mL) or without HQNO. The biofilm biomass was detected by a crystal violet microtiter plate assay. Error bars represent standard deviation from the mean value.

**Figure 4:**
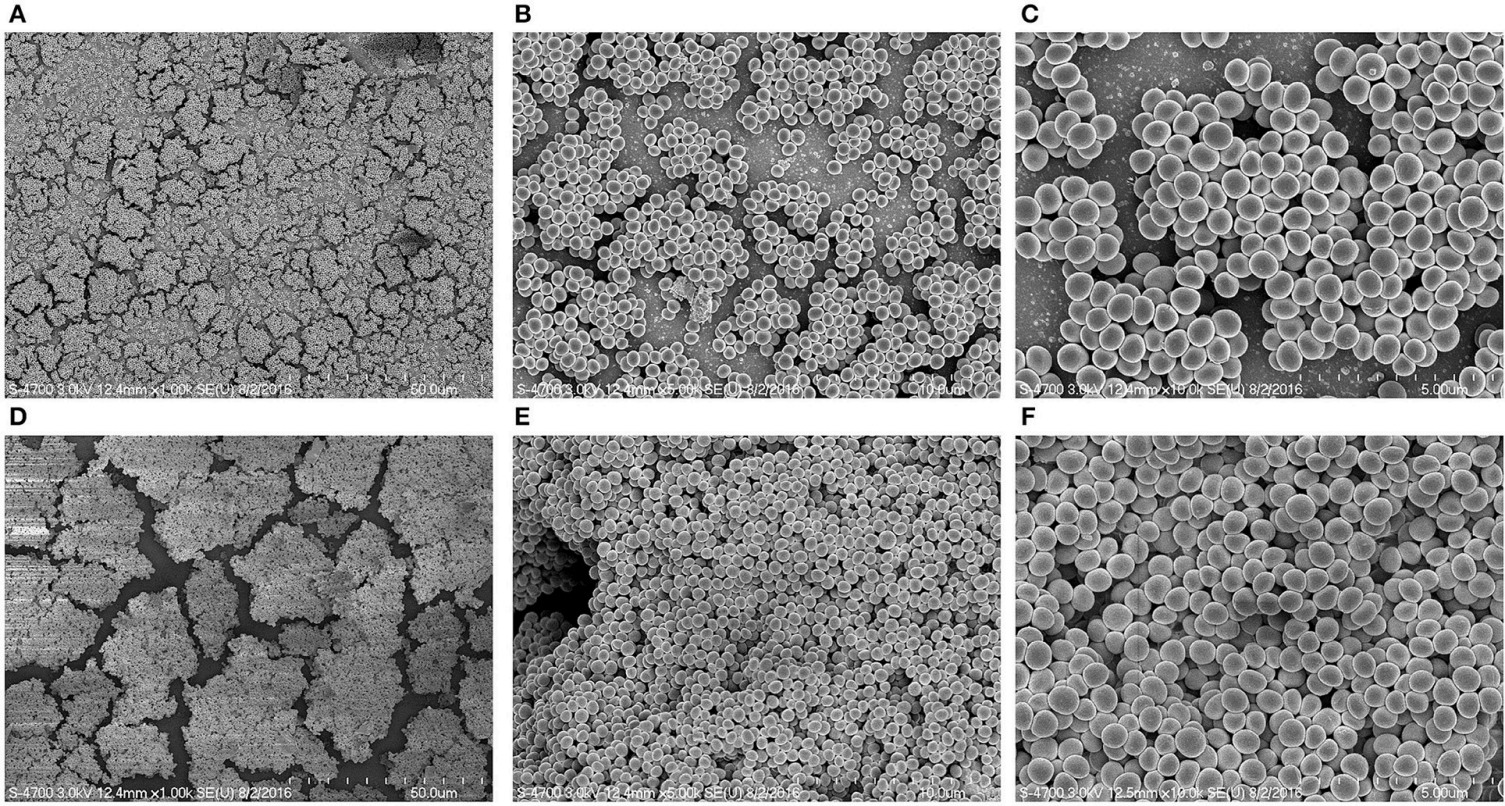
**Scanning electron microscopic (SEM) image of methicillin-resistant *Staphylococcus aureus* (MRSA) biofilms grown with and without HQNO**. Images **(A–C)** are representative SEM images of control biofilms (DMSO). **(D–F)** Representative SEM images of biofilm growth with 20 μg/ml HQNO. Scale bar = 5, 10, 50 μm.

The *in vitro* biofilm formation ability of MRSA 3903 SCVs was evaluated by culturing biofilms in the presence or absence of HQNO. CV-microtiter plate assay results showed no significant decrease in MRSA biofilm biomass in the presence of 20 μg HQNO (Figure 3). SEM images revealed no significant difference between SCV biofilms and phenotypically normal MRSA biofilms (Figure 4). These results indicate that MRSA 3903 could form SCVs, and that MRSA could exist in multi-species biofilms with PA.

### *In vivo* colonization of MRSA and PA alone or together in the rat middle ear

**Figure 5:**
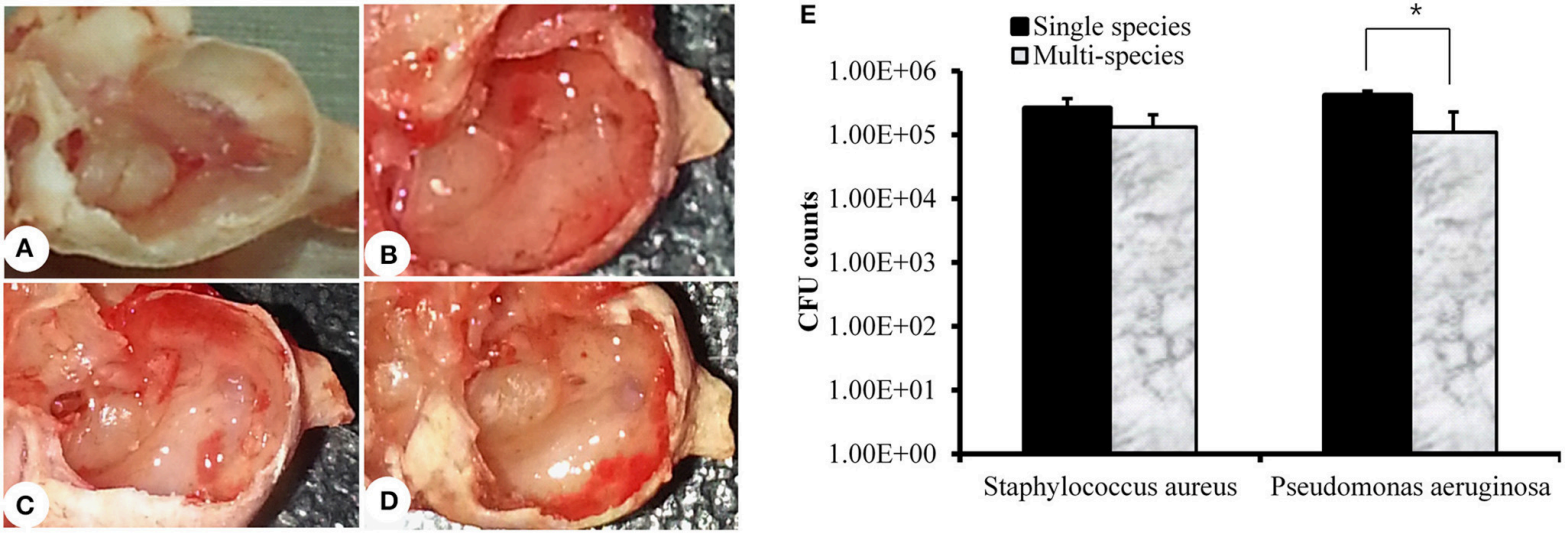
**Quantification of methicillin-resistant *Staphylococcus aureus* (MRSA) and *Pseudomonas aeruginosa* (PA) in the rat middle ear. (A)** Digital image of rat bulla inoculated with media only. **(B)** Digital image of rat bulla inoculated with MRSA only. **(C)** Digital images of rat bulla inoculated with PA only. **(D)** Digital image of rat bulla inoculated with multi-species (MRSA + PA). **(E)** Colony forming unit (CFU) counts for rat bullae inoculated with single or multi-species MRSA or PA in the rat middle ear.

Rats were sacrificed 1 week after inoculation, and bullae were acquired. The representative rat bullae were dissected and photographed (Figures 5A–D). The rat bullae inoculated with media only (vehicle control) were clear with no sign of mucosal inflammation or bacterial colonization (Figure 5A). However, the rat bullae inoculated with MRSA or PA alone or together were filled with a sticky fluid and showed signs of bacterial colonization. The mucosa was thick, swollen, and showed signs of severe inflammation (Figures 5B–D). The mean CFU counts for the rat bullae inoculated with MRSA and PA alone were 2.63 × 10^5^ (*SD* = 1.07 × 10^5^) and 4.50 × 10^5^ (*SD* = 7.07 × 10^4^), respectively. A higher number of PA CFUs, compared to MRSA CFUs, was recovered from rat bullae inoculated with either MRSA or PA alone; however, this difference was not significant. The CFU counts for MRSA and PA in the rat bullae inoculated with both species were 1.34 × 10^5^ (*SD* = 1.1 × 10^5^) and 1.10 × 10^5^ (*SD* = 7.3 × 10^4^), respectively (Figure 5E). No significant difference in CFU counts between MRSA and PA were detected in multi-species-inoculated rat bullae. However, the CFU counts for PA alone were significantly high than those of PA in multi-species inoculations.

### Visualization of *in vivo* colonization of MRSA and PA alone or in combination

**Figure 6:**
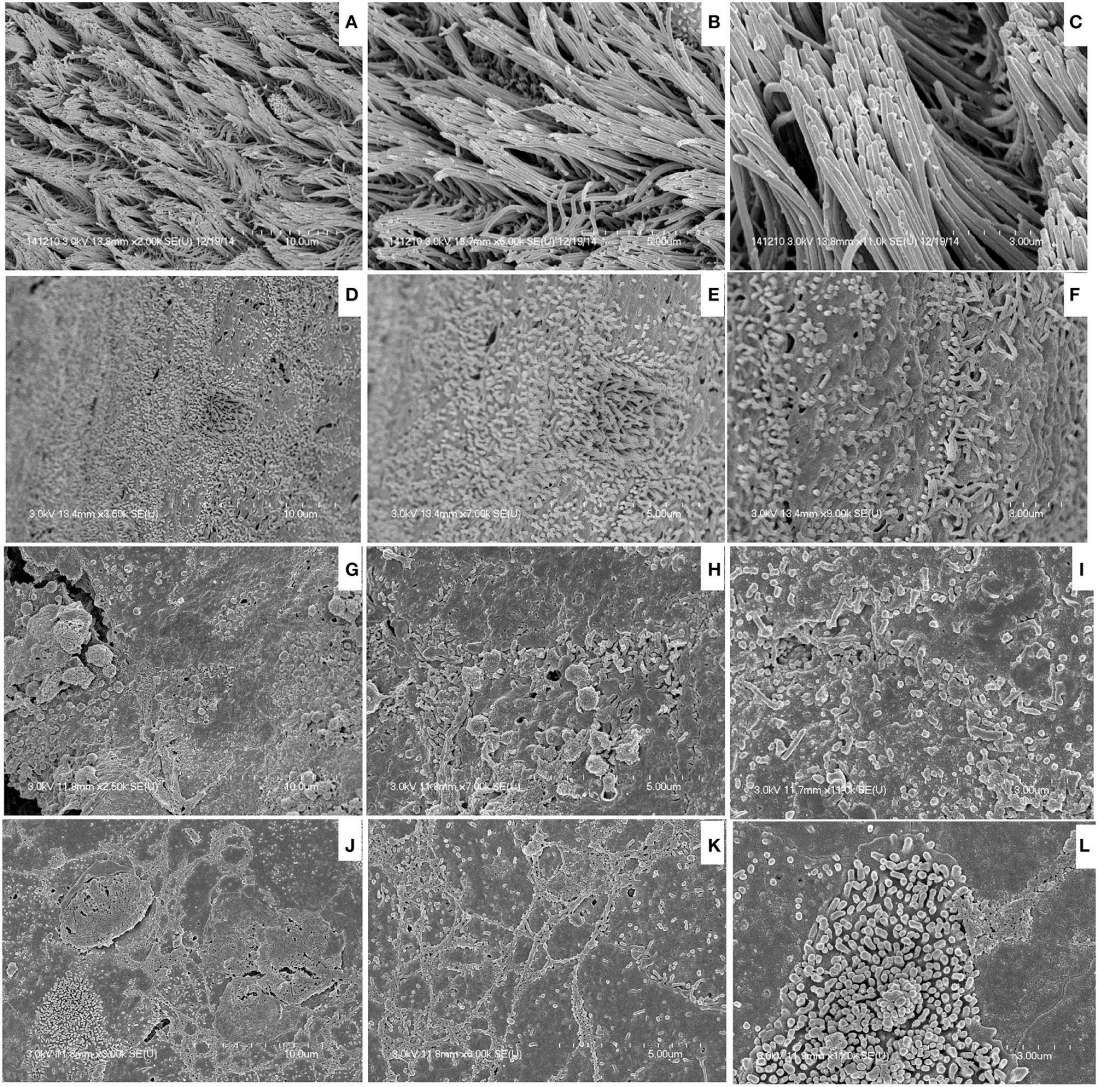
**Scanning electron microscope (SEM) images of bullae inoculated with methicillin-resistant *Staphylococcus aureus* (MRSA) and *Pseudomonas aeruginosa* (PA) as a single or multi-species inoculum. (A–C)** Representative SEM images of no procedure control. **(D–F)** Representative SEM images of rat bullae inoculated with SA only. **(G–I)** Representative SEM images of rat bullae inoculated with PA only. **(J–L)** Representative SEM images of rat bullae inoculated with a mixed culture of MRSA and PA.

SEM images of rat middle ears inoculated with MRSA, PA, MRSA + PA, or control inoculum are shown in Figure 6. In the control middle ear, the ciliated epithelium in the hypotympanum area and Eustachian tube orifice were intact (Figures 6A–C). In the MRSA, PA, or MRSA + PA treated groups, the entire middle ear was covered with cell debris or biofilm EPS. The tips of the cilia were invisible due to the deposition of cell debris (Figures 6D–L).

### Transcriptome sequencing of rat mucosa inoculated with MRSA, PA, or a combination of the two

**Figure 7:**
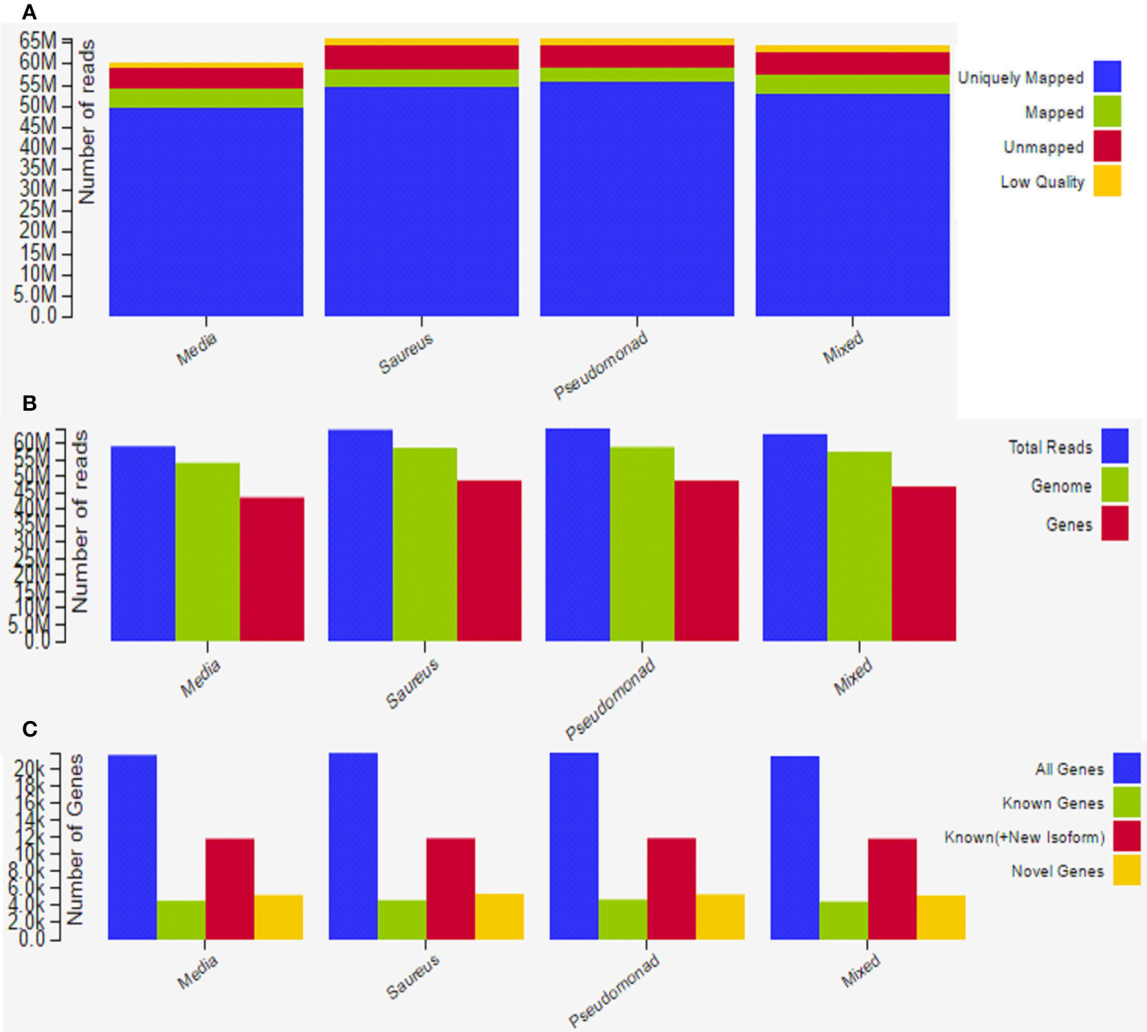
**Overview and summary of raw data analysis after transcriptome sequencing to assess gene expression in rat middle ear mucosae inoculated with methicillin-resistant *Staphylococcus aureus* (MRSA) or *Pseudomonas aeruginosa* (PA) as a single species or multi-species inoculum. (A)** Graphical representation of uniquely mapped, mapped, and unmapped reads. **(B)** Graphical representation of total reads, gene reads, and genome reads. **(C)** Graphical representation of novel genes, known genes, and all genes.

Total raw data reads in all samples ranged from 50 to 65 million, with an average of approximately 55 million raw reads per sample. The average mRNA insert size detected by sequencing and mapping was 159 bp. The uniquely mapped reads ranged between ~50 and 55 million; there were ~5 million mapped reads and ~6 million unmapped reads (Figure 7A). There were ~58 million total reads, 55 million genome reads, and 43 million gene reads (Figure 7B). The gene reads were ~20 K, known genes were ~5 K, known plus new isoforms were ~12 K, and novel genes were ~5 K (Figure 7C). A total of 1797 genes were significantly (*p* < 0.05) differentially expressed in all three treatments with respect to the control (media). Among them, 973 were upregulated > 1-fold and 824 were downregulated > 1-fold.

### Differential gene expression

**Figure 8:**
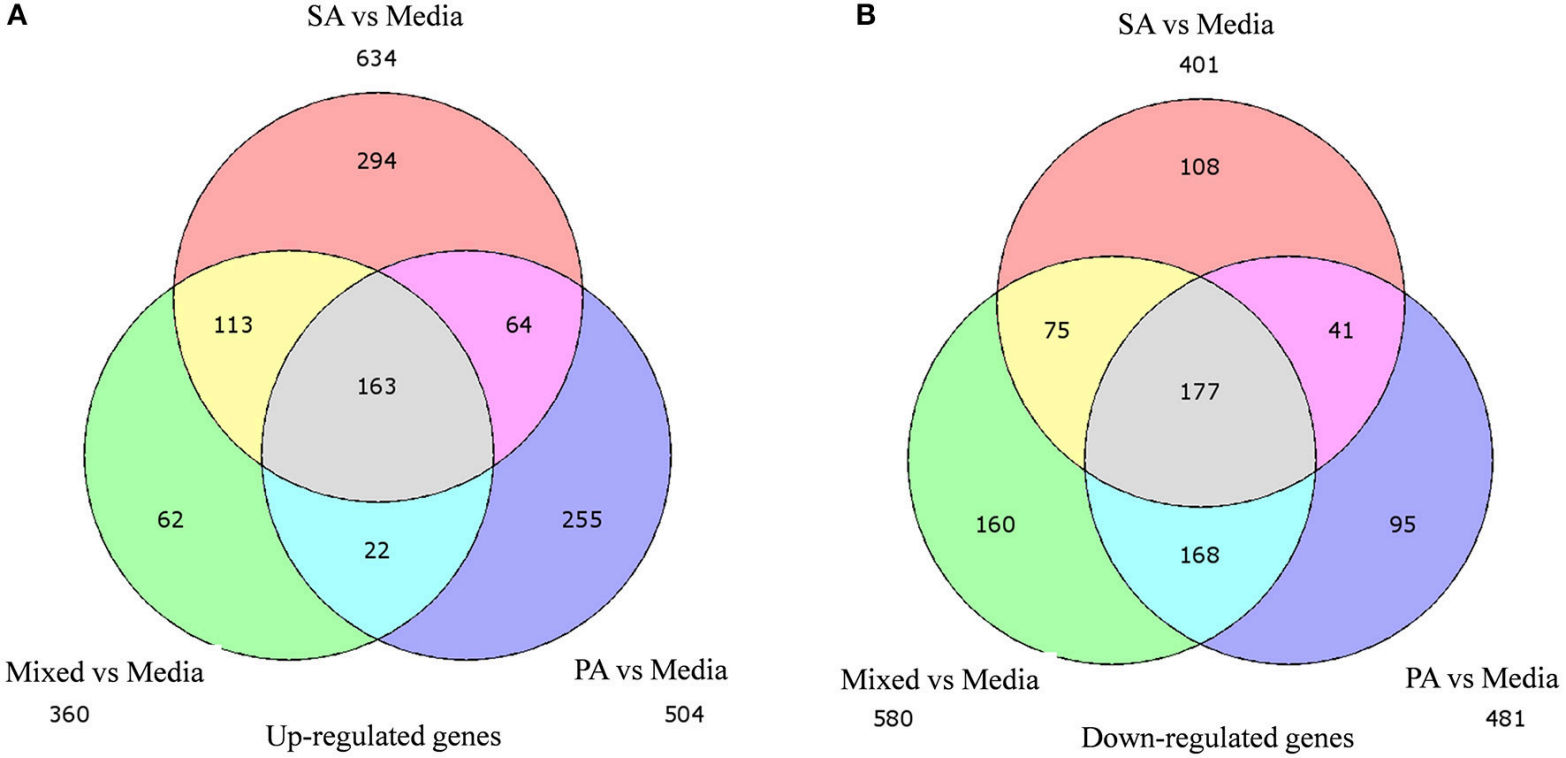
**Venn diagram illustrating the number of genes commonly or uniquely differentially expressed in rat middle ear mucosa colonized with methicillin-resistant *Staphylococcus aureus* (MRSA) or *Pseudomonas aeruginosa* (PA) as a single species or multi-species inoculum. (A)** Venn diagram showing significantly (*p* < 0.05) upregulated genes after colonization with MRSA, PA, or a combination of the two, with respect to the media (control). **(B)** Venn diagram showing significantly (*p* < 0.05) downregulated genes after MRSA, PA, or multi-species colonization, with respect to media control.

**Figure 9:**
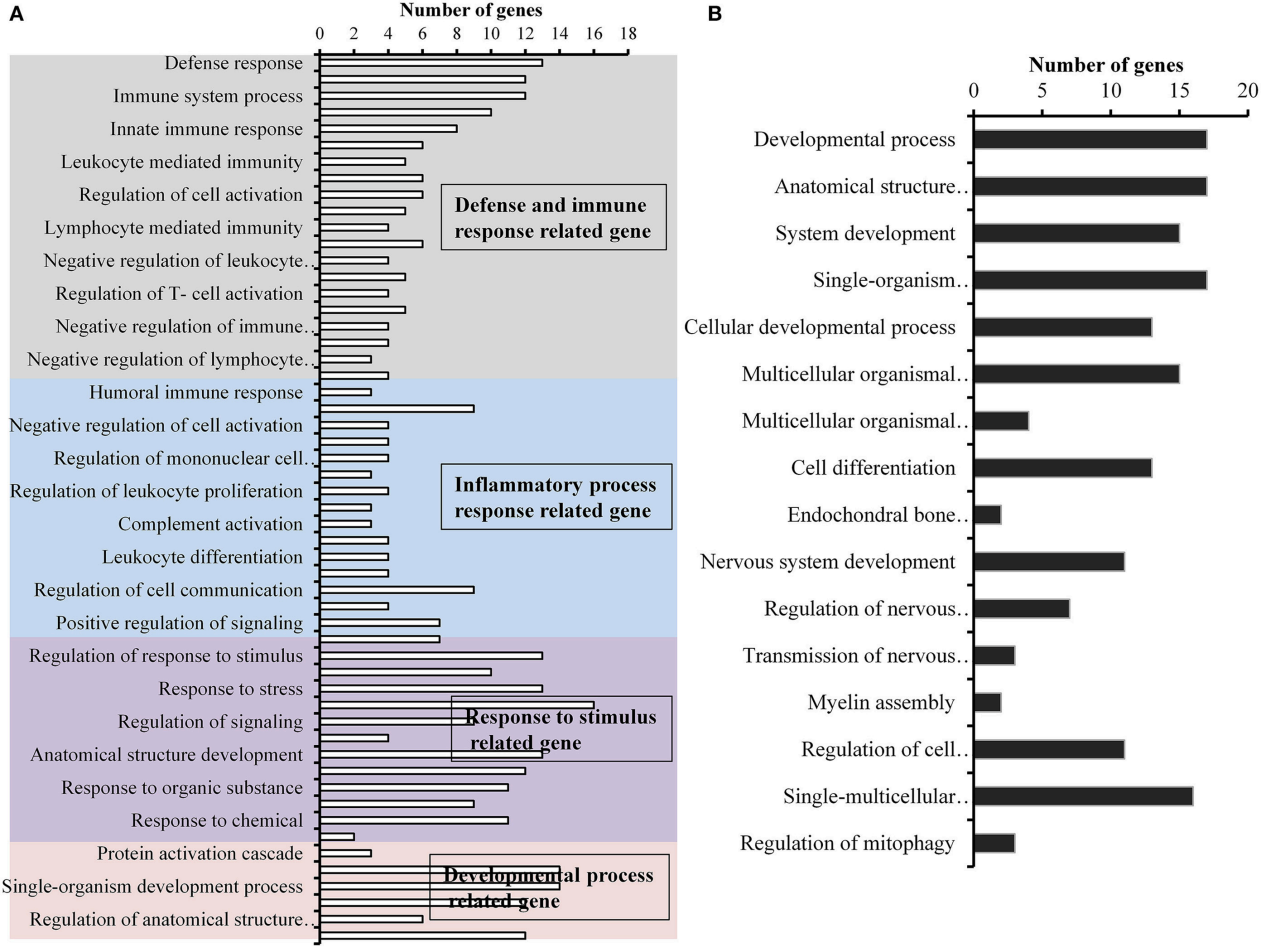
**Gene ontology (GO) of differentially expressed genes in rat middle ear mucosa colonized with methicillin-resistant *Staphylococcus aureus* (MRSA) or *Pseudomonas aeruginosa* (PA) as a single or multi-species inoculum. (A)** GO category of significantly (*p* < 0.05) upregulated genes (by ≥1.5-fold) with multi-species colonization. **(B)** GO category of significantly (*p* < 0.05) downregulated genes (≥1.5-fold) after multi-species colonization.

**Figure 10:**
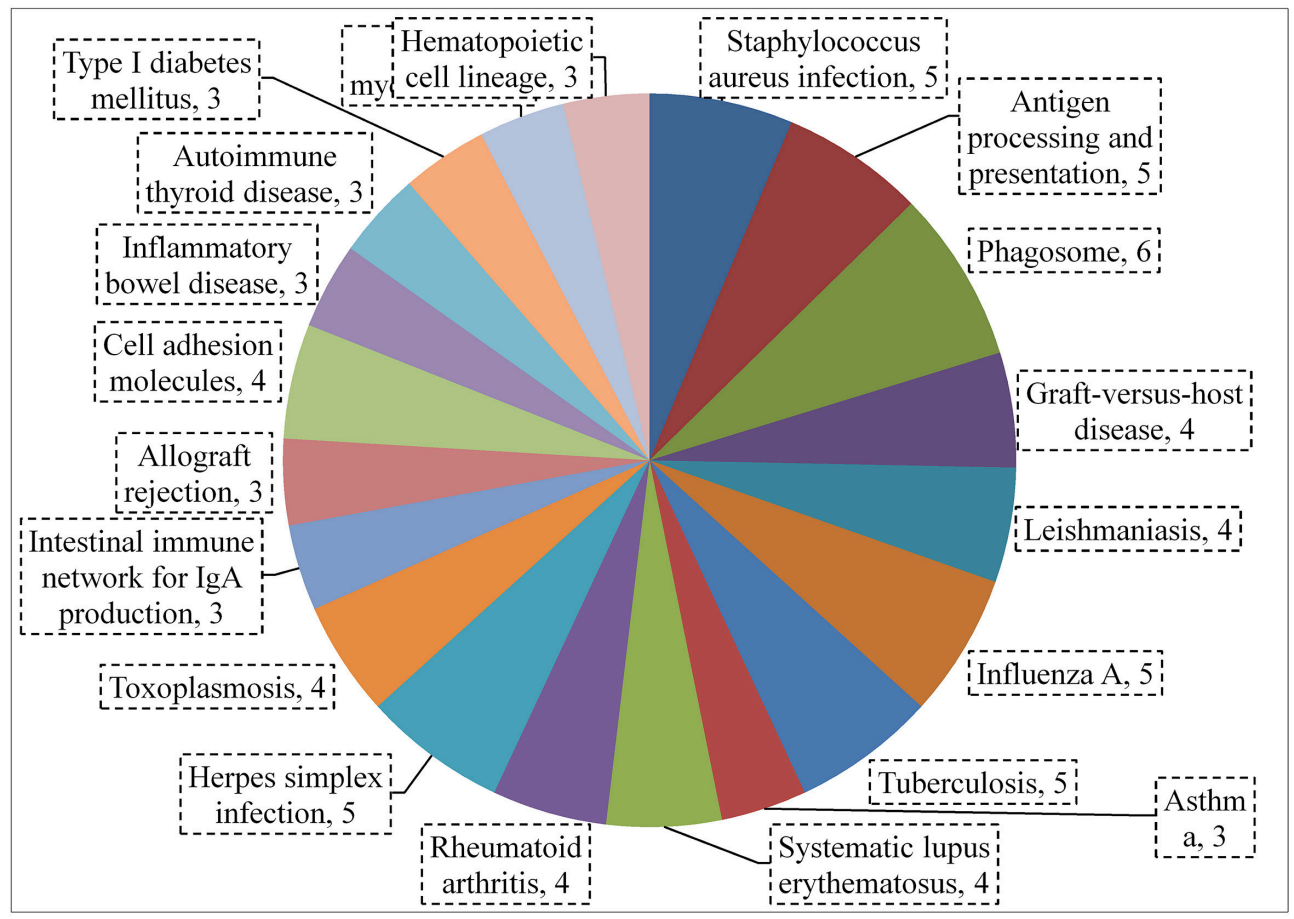
**KEGG pathway analysis using string showing significantly (*p* < 0.05) upregulated genes (by ≥1.5-fold) after multi-species colonization of the rat middle ear mucosa**.

A total of 360, 634, and 504 genes were significantly (*p* < 0.05) upregulated in multi-species, MRSA and PA, colonized rat middle ear mucosae (Figure 8A). Among 360 upregulated genes in multi-species-colonized rat mucosae, 113 (31%) were similar to those of MRSA, and 22 (6.1%) were similar to those of PA. The three treatments shared 163 genes whose expression was upregulated. Moreover, 62 genes were exclusively upregulated during multi-species colonization. Among them, the 42 genes upregulated by ≥1.5-fold and encoding proteins of known functions are shown in Table 2. A total of 580, 401, and 481 genes were significantly (*p* < 0.05) downregulated in multi-species-, MRSA-, and PA-colonized rat middle ear mucosae (Figure 8B). Among the 580 downregulated genes after multi-species colonization, 75 genes (12.9%) were similar to those associated with MRSA, and 168 (28.9%) genes were similar to those associated with PA. Among 160 genes exclusively downregulated after multi-species colonization, 88 genes that were downregulated by ≥1.5-fold and encoding known proteins are shown in Table 3. The gene ontology (GO) of upregulated and downregulated genes in multi-species inoculated rat mucosae is shown in Figures 9A,B. The GO analysis of 42 protein-encoding genes that were upregulated (≥1.5-fold) after multi-species colonization revealed that genes involved in defense, immune response, inflammatory response, and developmental process were exclusively upregulated after multi-species inoculation (Figure 9A). The GO analysis of 88 protein-encoding genes that were downregulated (≥1.5-fold) revealed that genes that are involved in nervous system signaling, development and transmission, regulation of cell growth and development, anatomical and system development, and cell differentiation were significantly downregulated after multi-species inoculation (Figure 9B). Based on KEGG pathway analysis of upregulated genes after multi-species inoculation, genes involved in phagosome, antigen processing and presentation, influenza A infection, *Staphylococcus* infection, herpes virus infection, toxoplasmosis, and tuberculosis (and other) pathway genes were evaluated (Figure 10). These results revealed that colonization of the rat middle ear with MRSA or PA alone or in combination regulates a significant number of genes. In addition, multi-species colonization with MRSA and PA induced a different set of genes that were not induced by either MRSA or PA alone. This shows that the host response is different after multi-species colonization, which could enhance the pathogenicity of the bacteria.

**Table 2:** **List of genes upregulated ≥1.5-fold (*p* < 0.05) in rat middle ear mucosae colonized with MRSA and PA and upregulated or downregulated with MRSA or PA single species colonization**.

**Serial number**	**Gene accession**	**Gene ID**	**Gene name**	**Protein**	**Fold change in mixed inoculation**	***p*****-value**	**Fold change with single species SA**	***p*****-value**	**Fold change with single species PA**	***p*****-value**
1	XLOC_007451	TBIG007451	*CHI3L1*	Chitinase-3-like protein 1	1.5	0.0127	1.35	0.0061	1.21	0.0636
2	XLOC_008245	TBIG008245	*PPP2R3D*	Serine/threonine-protein phosphatase 2A regulatory subunit B” subunit delta	1.52	0.0412	1.67	0.00865	1.38	0.08015
3	XLOC_010172	TBIG010172	*JAK3*	Tyrosine-protein kinase JAK3	1.52	0.01095	1.31	0.01205	1.64	0.0179
4	XLOC_016832	TBIG016832	*FCN2*	Ficolin-2	1.53	0.04235	0.547	0.38185	0.917	0.2557
5	XLOC_015362	TBIG015362	*RT1-BA*	Rano class II histocompatibility antigen, B alpha chain	1.54	0.0146	1.42	0.00855	0.573	0.35145
6	XLOC_026947	TBIG026947	*TLR7*	Toll-like receptor 7	1.55	0.0207	1.68	0.0028	1.4	0.0428
7	XLOC_023774	TBIG023774	*SLA*	Src-like-adapter	1.55	0.01355	1.65	0.0038	1.47	0.04885
8	XLOC_002817	TBIG002817	*CORO1A*	Coronin-1A	1.56	0.0055	1.5	0.00155	1.43	0.019
9	XLOC_014051	TBIG014051	*CFI*	Complement factor I	1.57	0.04835	0.89	0.1797	1.96	0.0404
10	XLOC_017708	TBIG017708	*CTSZ*	Cathepsin Z	1.57	0.0143	1.36	0.0164	0.876	0.19125
11	XLOC_013234	TBIG013234	*CDH6*	Cadherin-6	1.61	0.0029	1.22	0.00625	2.64	5.00*E*−05
12	XLOC_009200	TBIG009200	*DNASE1L3*	Deoxyribonuclease gamma	1.63	0.0323	1.61	0.01495	0.767	0.3716
13	XLOC_004746	TBIG004746	*PRSS22*	Brain-specific serine protease 4	1.66	0.0247	0.698	0.2435	2.12	0.0144
14	XLOC_025304	TBIG025304	*RPLP1*	60S acidic ribosomal protein P1	1.67	0.00535	1.6	0.00365	0.472	0.41575
15	XLOC_024595	TBIG024595	*STRA6*	Stimulated by retinoic acid gene 6 protein homolog	1.67	0.0355	0.526	0.4289	2.35	0.011
16	XLOC_026676	TBIG026676	*ST8SIA4*	CMP-N-acetylneuraminate-poly-alpha-2,8-sialyltransferase	1.67	0.0333	1.43	0.02115	2.04	0.01315
17	XLOC_006363	TBIG006363	*TFRC*	Transferrin receptor protein 1	1.69	0.0043	0.726	0.1144	0.124	0.82765
18	XLOC_016540	TBIG016540	*HCK*	Tyrosine-protein kinase HCK	1.7	0.00685	1.94	0.00045	1.36	0.04235
19	XLOC_006164	TBIG006164	*SAMSN1*	SAM domain-containing protein SAMSN-1	1.71	0.0231	1.8	0.00595	1.34	0.09725
20	XLOC_019232	TBIG019232	*ENV*	Envelope glycoprotein gp70	1.71	0.0325	1.3	0.0364	1.87	0.0289
21	XLOC_007736	TBIG007736	*LAMB3*	Laminin subunit beta-3	1.71	0.0046	1.01	0.0415	2.27	0.0012
22	XLOC_014992	TBIG014992	*RT1-BB*	Rano class II histocompatibility antigen, B-1 beta chain	1.72	0.0213	1.54	0.0081	0.402	0.48745
23	XLOC_014993	TBIG014993	*RT1-DB1*	Rano class II histocompatibility antigen, D-1 beta chain	1.74	0.00385	1.59	0.00255	1.3	0.0427
24	XLOC_020229	TBIG020229	*CNR2*	Cannabinoid receptor 2	1.74	0.04435	1.69	0.0167	1.57	0.12025
25	XLOC_002804	TBIG002804	*LAT*	Linker for activation of T-cells family member 1	1.77	0.0202	1.37	0.0229	1.34	0.10215
26	XLOC_016951	TBIG016951	*RPS17*	40S ribosomal protein S17	1.8	0.04525	1.42	0.06125	−0.406	0.725
27	XLOC_023263	TBIG023263	*KRT7*	Keratin, type II cytoskeletal 7	1.81	0.003	0.839	0.10965	2.18	0.00285
28	XLOC_025817	TBIG025817	*C3*	Complement C3	1.85	0.0068	1.81	0.00065	0	1
29	XLOC_015648	TBIG015648	*ELL3*	RNA polymerase II elongation factor ELL3	1.9	0.04245	1.08	0.12345	0.566	0.57055
30	XLOC_002984	TBIG002984	*GAL*	Galanin peptides	1.91	0.03065	0.656	0.36215	−0.583	0.604
31	XLOC_016373	TBIG016373	*NMES1*	Normal mucosa of esophagus-specific gene 1 protein	2.04	0.01855	1.79	0.0102	1.43	0.1201
32	XLOC_018558	TBIG018558	*KLRD1*	Natural killer cells antigen CD94	2.07	0.0351	1.83	0.05635	0.31	0.8115
33	XLOC_001187	TBIG001187	*SRCRM_HUMAN*	Putative scavenger receptor cysteine-rich domain-containing protein LOC619207	2.07	0.0019	2.57	5.00*E*−05	0.401	0.5927
34	XLOC_018355	TBIG018355	*CD8A*	T-cell surface glycoprotein CD8 alpha chain	2.11	0.0217	2.51	0.00255	1.2	0.23315
35	XLOC_020260	TBIG020260	*PLA2G2D*	Group IID secretory phospholipase A2	2.25	0.01785	1.44	0.0665	0.51	0.6369
36	XLOC_023454	TBIG023454	*GZMM*	Granzyme M	2.41	0.0116	2.33	0.0055	−0.876	0.44375
37	XLOC_005212	TBIG005212	*CCL5*	C-C motif chemokine 5	2.42	0.01385	2.05	0.00985	−0.559	0.6301
38	XLOC_022420	TBIG022420	*HVM17_MOUSE*	Ig heavy chain V region MOPC 47A	2.51	0.02705	1.16	0.22905	0.871	0.51305
39	XLOC_015317	TBIG015317	*UBD*	Ubiquitin D	2.7	0.00275	1.93	0.00665	1.14	0.2022
40	XLOC_011426	TBIG011426	*TCC2_MOUSE*	T-cell receptor gamma chain C region C7.5	2.72	0.0168	3.31	0.00035	0.352	0.78555
41	XLOC_006104	TBIG006104	*LV2B_MOUSE*	Ig lambda-2 chain V region MOPC 315	2.8	0.002	0.785	0.2881	0.937	0.33275
42	XLOC_018648	TBIG018648	*KLRB1F*	Killer cell lectin-like receptor subfamily B member 1F	2.82	0.02	2.45	0.02085	0.0885	0.9476
43	XLOC_012586	TBIG012586	*CLEC3A*	C-type lectin domain family 3 member A	3.07	0.04855	3.67	0.014	1.08	0.46495
44	XLOC_022400	TBIG022400	*HVM43_MOUSE*	Ig heavy chain V region MOPC 141	3.15	0.04835	3.26	0.06595	0.34	0.8163

**Table 3:** **List of genes downregulated by ≥1.5-fold (*p* < 0.05) in rat middle ear mucosae colonized by MRSA and PA and either downregulated or upregulated with MRSA or PA single species colonization**.

**Serial number**	**Gene accession**	**Gene ID**	**Gene name**	**Protein**	**Fold change in mixed inoculation**	***p*****-value**	**Fold change with single species SA**	***p*****-value**	**Fold change with single species PA**	***p*****-value**
1	XLOC_000033	TBIG000033	*TXLNB*	Beta-taxilin	−7.41	0.04245	−1.39	0.01125	−7.68	0.07995
2	XLOC_005477	TBIG005477	*SCN4A*	Sodium channel protein type 4 subunit alpha	−5.49	0.02615	−1.34	0.074	−4.26	0.11755
3	XLOC_021210	TBIG021210	*GAG*	Gag polyprotein	−5.22	0.044	0.78	0.2248	−0.722	0.4373
4	XLOC_027720	TBIG027720	*IGSF1*	Immunoglobulin superfamily member 1	−4.53	0.0182	−1.13	0.19385	−5.06	0.14935
5	XLOC_013130	TBIG013130	*LDLRAD1*	Low-density lipoprotein receptor class A domain-containing protein 1	−4.36	0.0083	−1.15	0.2058	−1.93	0.15705
6	XLOC_027382	TBIG027382	*POL*	Retrovirus-related Pol polyprotein LINE-1	−4.29	0.0183	−1.76	0.07165	−4.89	0.095
7	XLOC_012347	TBIG012347	*GAG-POL*	Gag-Pol polyprotein	−4.25	0.00015	0.841	0.0705	−0.867	0.15245
8	XLOC_015004	TBIG015004	*TNXB*	Tenascin-X	−4.14	0.0376	−2.21	0.25835	−3	0.0938
9	XLOC_024947	TBIG024947	*GRIA4*	Glutamate receptor 4	−4.06	0.04955	−1.7	0.0813	−2.26	0.1075
10	XLOC_014321	TBIG014321	*TTC24*	Tetratricopeptide repeat protein 24	−4.04	0.0285	−0.657	0.4197	−1.2	0.28155
11	XLOC_026757	TBIG026757	*POL*	Pol polyprotein	−4.03	0.02135	0.779	0.21675	1.18	0.15825
12	XLOC_020111	TBIG020111	*NT5C1A*	Cytosolic 5'-nucleotidase 1A	−3.93	0.00125	−1.39	0.0508	−2.43	0.0548
13	XLOC_002039	TBIG002039	*MARK4*	MAP/microtubule affinity-regulating kinase 4	−3.65	0.0475	−1.43	0.4849	−3.48	0.0536
14	XLOC_007497	TBIG007497	*BRINP3*	BMP/retinoic acid-inducible neural-specific protein 3	−3.63	0.0429	−1.46	0.1324	−3.66	0.13615
15	XLOC_024201	TBIG024201	*POL*	Pol polyprotein	−3.57	0.0373	−0.937	0.2889	−1.6	0.2579
16	XLOC_006129	TBIG006129	*POL*	Pol polyprotein	−3.38	0.04745	−1.18	0.207	−1.52	0.34225
17	XLOC_005475	TBIG005475	*GH1*	Somatotropin	−3.34	0.04165	0.851	0.2985	−0.925	0.49845
18	XLOC_024602	TBIG024602	*HCN4*	Potassium/sodium hyperpolarization-activated cyclic nucleotide-gated channel 4	−3.27	0.03825	−0.742	0.37845	−1.53	0.27585
19	XLOC_021698	TBIG021698	*RGS6*	Regulator of G-protein signaling 6	−3.25	0.02615	−1.38	0.14185	−1.96	0.20475
20	XLOC_006065	TBIG006065	*OSTN*	Osteocrin	−3.23	0.04325	−1.79	0.04165	−2.8	0.081
21	XLOC_021434	TBIG021434	*ALK*	ALK tyrosine kinase receptor	−3.18	0.0397	−1.86	0.0675	−3.07	0.08475
22	XLOC_026950	TBIG026950	*EGFL6*	Epidermal growth factor-like protein 6	−3.13	0.0404	−0.986	0.2498	−1.45	0.2599
23	XLOC_021001	TBIG021001	*NCMAP*	Noncompact myelin-associated protein	−3.13	0.0497	−1.11	0.50945	−1.23	0.5089
24	XLOC_004000	TBIG004000	*GRIA1*	Glutamate receptor 1	−3.05	0.0488	−2.95	0.0636	−2.59	0.0773
25	XLOC_021987	TBIG021987	*KCNK3*	Potassium channel subfamily K member 3	−2.98	0.03695	−2.13	0.0539	−2.07	0.1183
26	XLOC_008308	TBIG008308	*NAA11*	N-alpha-acetyltransferase 11	−2.97	0.02785	−1.27	0.1347	−1.53	0.1725
27	XLOC_003922	TBIG003922	*TRIM7*	Tripartite motif-containing protein 7	−2.93	0.0133	−1.02	0.11075	−1.47	0.10675
28	XLOC_010040	TBIG010040	*POL*	Pol polyprotein	−2.86	0.01455	−1.46	0.06215	−2.43	0.0726
29	XLOC_017690	TBIG017690	*BCAS1*	Breast carcinoma-amplified sequence 1 homolog	−2.65	0.04765	−1.54	0.07675	−0.974	0.3948
30	XLOC_002797	TBIG002797	*SNRPN*	Small nuclear ribonucleoprotein-associated protein N	−2.65	0.0201	−1.4	0.06855	−2.02	0.06625
31	XLOC_023030	TBIG023030	*COL14A1*	Collagen alpha-1(XIV) chain	−2.62	0.0069	−0.904	0.16355	−1.16	0.15975
32	XLOC_023753	TBIG023753	*KLHL38*	Kelch-like protein 38	−2.62	0.0302	−0.989	0.17215	−2.69	0.0851
33	XLOC_002608	TBIG002608	*HBB2_RAT*	Hemoglobin subunit beta-2	−2.55	0.0001	−2.36	5.00*E*−05	−3.34	5.00*E*−05
34	XLOC_024012	TBIG024012	*COL2A1*	Collagen alpha-1(II) chain	−2.51	0.00715	−0.616	0.24645	1.09	0.10775
35	XLOC_007817	TBIG007817	*NFASC*	Neurofascin	−2.46	0.02025	−1.47	0.09665	−2.29	0.08025
37	XLOC_018133	TBIG018133	*MEST*	Mesoderm-specific transcript homolog protein	−2.43	0.03235	−1.11	0.1112	−1.91	0.07395
38	XLOC_022758	TBIG022758	*LINGO3*	Leucine-rich repeat and immunoglobulin-like domain-containing nogo receptor-interacting protein 3	−2.35	0.0387	−1.42	0.0885	−1.4	0.18525
39	XLOC_005055	TBIG005055	*CHRNB1*	Acetylcholine receptor subunit beta	−2.25	0.0174	−1.12	0.10445	−1.7	0.08505
40	XLOC_009184	TBIG009184	*CDHR3*	Cadherin-related family member 3	−2.25	0.04445	−0.978	0.13985	−1.47	0.15415
41	XLOC_014711	TBIG014711	*CAPSL*	Calcyphosin-like protein	−2.24	0.04895	−0.886	0.25825	−0.976	0.37135
42	XLOC_003432	TBIG003432	*POL*	Pol polyprotein	−2.11	0.0243	−1.35	0.0438	−1.65	0.07025
43	XLOC_022093	TBIG022093	*EGLN3*	Egl nine homolog 3	−2.09	0.02785	−0.962	0.11	−0.67	0.37785
44	XLOC_017633	TBIG017633	*MATN4*	Matrilin-4 [Source:SWISS;Acc:O89029]	−2.02	0.0068	−0.87	0.0838	−0.925	0.15585
45	XLOC_017934	TBIG017934	*GAG-POL*	Gag-Pol polyprotein	−1.92	0.0217	−0.381	0.4882	−0.885	0.2449
46	XLOC_000295	TBIG000295	*SSC5D*	Soluble scavenger receptor cysteine-rich domain-containing protein SSC5D	−1.91	0.00235	−0.86	0.062	−1.13	0.05595
47	XLOC_020236	TBIG020236	*TCEA3*	Transcription elongation factor A protein 3	−1.8	0.04715	−0.75	0.2413	−1.45	0.1176
48	XLOC_019566	TBIG019566	*POL*	Pol polyprotein	−1.75	0.00645	−0.592	0.29525	−1.36	0.0589
49	XLOC_024924	TBIG024924	*FAM198A*	Protein FAM198A	−1.68	0.04485	−0.975	0.104	−1.57	0.0906
50	XLOC_016802	TBIG016802	-	-	−1.68	0.01715	−0.262	0.6802	−1.08	0.13725
51	XLOC_010859	TBIG010859	*AGTPBP1*	Cytosolic carboxypeptidase 1	−1.67	0.01575	−0.817	0.1006	−1.66	0.0367
52	XLOC_002382	TBIG002382	*ST8SIA2*	Alpha-2,8-sialyltransferase 8B	−1.67	0.04965	−0.846	0.171	−1.09	0.23515
53	XLOC_026579	TBIG026579	*CCDC108*	Coiled-coil domain-containing protein 108	−1.66	0.01635	−0.31	0.5204	−0.735	0.2972
54	XLOC_027640	TBIG027640	*CHRDL1*	Chordin-like protein 1	−1.62	0.00485	−0.842	0.0639	−1.81	0.0037
55	XLOC_002898	TBIG002898	*ADAM8*	Disintegrin and metalloproteinase domain-containing protein 8	−1.59	0.00935	−0.908	0.0545	−0.97	0.12135
56	XLOC_021028	TBIG021028	*UBXN10*	UBX domain-containing protein 10	−1.58	0.01805	−0.353	0.5061	−1.22	0.08675
57	XLOC_013884	TBIG013884	*C1ORF173*	Uncharacterized protein C1orf173	−1.56	0.019	−0.068	0.88615	−0.855	0.18325
58	XLOC_024898	TBIG024898	*DLEC1*	Deleted in lung and esophageal cancer protein 1	−1.56	0.03495	−0.467	0.3517	−1.07	0.14125
59	XLOC_001829	TBIG001829	*SLC22A3*	Solute carrier family 22 member 3	inf	5.00*E*−05	−1.36	0.2606	−3.68	0.19145
60	XLOC_003476	TBIG003476	*POL*	Pol polyprotein	inf	5.00*E*05	−0.582	0.23855	−10.4	0.2431
61	XLOC_004811	TBIG004811	*RPS2*	40S ribosomal protein S2	inf	5.00*E*−05	0.17	0.71865	inf	5.00*E*−05
62	XLOC_006114	TBIG006114	*RPL9*	60S ribosomal protein L9	inf	5.00*E*−05	−0.538	0.5414	inf	5.00*E*−05
63	XLOC_006289	TBIG006289	*MYH15*	Myosin-15	inf	5.00*E*−05	−1.09	0.28855	−6.22	0.2431
64	XLOC_006508	TBIG006508	*GAG*	Gag polyprotein	inf	5.00*E*−05	−0.187	0.8494	−3.29	0.21805
65	XLOC_009108	TBIG009108	*GAG-POL*	Gag-Pol polyprotein	inf	5.00*E*−05	−0.5	0.5663	−6.64	0.24325
66	XLOC_013224	TBIG013224	*RPS2*	40S ribosomal protein S2	inf	5.00*E*−05	0.11	0.8856	−7.12	0.24315
67	XLOC_017320	TBIG017320	*ACTC1*	Actin, alpha cardiac muscle 1	inf	5.00*E*−05	−0.929	0.0515	−11.1	0.2431
68	XLOC_022977	TBIG022977	*STAC3*	SH3 and cysteine-rich domain-containing protein 3	inf	5.00*E*−05	−1.31	0.0224	−8.87	0.1708
69	XLOC_023915	TBIG023915	*CYP2D1*	Cytochrome P450 2D1	inf	5.00*E*−05	1.29	0.09275	−4.64	0.16405
70	XLOC_026493	TBIG026493	*MSTN*	Growth/differentiation factor 8	inf	5.00*E*−05	−2.2	0.1506	−5.46	0.2456
71	XLOC_027253	TBIG027253	*CX064_MOUSE*	Uncharacterized protein CXorf64 homolog	inf	5.00*E*−05	−1.48	0.1895	−5.75	0.2454
72	XLOC_022618	TBIG022618	*ENV*	Envelope glycoprotein	inf	0.0004	−0.993	0.41135	inf	0.0008
73	XLOC_005431	TBIG005431	*NEDD4*	E3 ubiquitin-protein ligase NEDD4	inf	0.00345	−1.81	0.2551	−4.4	0.25485
74	XLOC_016973	TBIG016973	*NEB*	Nebulin	inf	0.00495	−0.73	0.57485	−2.83	0.2479
75	XLOC_019744	TBIG019744	*CALB1*	Calbindin	inf	0.0053	−1.73	0.2773	−3.48	0.2558
76	XLOC_020636	TBIG020636	*15.5 KDA FABP*	Major urinary protein	inf	0.0053	−3.54	0.1979	−2.95	0.2434
77	XLOC_022462	TBIG022462	*HV208_HUMAN*	Ig heavy chain V-II region SESS	inf	0.0053	−0.717	0.578	−4.71	0.2508
78	XLOC_027795	TBIG027795	*POL*	Pol polyprotein	inf	0.0053	−3	0.1555	−3.64	0.25175
79	XLOC_007006	TBIG007006	*KIF19*	Kinesin-like protein KIF19	inf	0.0058	−1.9	0.2472	−4.39	0.2531
80	XLOC_017904	TBIG017904	*POL*	Retrovirus-related Pol polyprotein LINE-1	inf	0.00575	−3.28	0.2009	−1.76	0.3774
81	XLOC_019217	TBIG019217	*OXTR*	Oxytocin receptor	inf	0.0058	−2.29	0.19495	−4.46	0.2524
82	XLOC_007150	TBIG007150	*DNAH10*	Dynein heavy chain 10, axonemal	inf	0.01035	−3.34	0.26695	−2.62	0.2963
83	XLOC_016972	TBIG016972	*NEB*	Nebulin	inf	0.0097	−2.55	0.23375	−3.61	0.2679
84	XLOC_009140	TBIG009140	*DUSP13*	Dual specificity protein phosphatase 13 isoform A	inf	0.0117	−0.865	0.6321	−3.34	0.27455
85	XLOC_006921	TBIG006921	*CD209L2*	CD209 antigen-like protein 2	inf	0.01335	−0.951	0.6269	−3.07	0.2863
86	XLOC_009994	TBIG009994	*GAG*	Gag polyprotein	inf	0.01335	1.01	0.46895	0.352	0.8164
87	XLOC_009143	TBIG009143	*DUSP13*	Dual specificity protein phosphatase 13 isoform A	inf	0.0252	−0.929	0.6643	−2.67	0.42285
88	XLOC_007324	TBIG007324	*POL*	Pol polyprotein	inf	0.0387	0.404	0.83325	0.731	0.77005

### Quantification of gene expression using real-time RT-PCR

The results of real time-PCR were in complete agreement with RNA sequencing gene expression results. The expression of *RPS9, MMP12, ITGB2, CST7, SCTR, APOC1, CD69*, and *CCNO* genes was significantly (*p* < 0.05) upregulated (by >1.5-fold) in rat mucosae colonized with MRSA or PA alone or in combination based on both real time RT-PCR and RNA sequencing (Table 4). Similarly, the expression of *KERA, GPD1, ACTA1, EEF1A2, HRC, FOXD1, MYH1, SYNPO2L, TNMD*, and *MYOM2*, based on real time RT-PCR and RNA sequencing, was significantly (*p* < 0.05) decreased by more than 1.5-fold (Table 4).

**Table 4:** **Confirmation of RNA sequencing results by real-time RT-PCR**.

	**Gene name**	**Rat colonized with multi-species culture**		**Rat colonized with MRSA single species**		**Rat colonized with PA single species**	
		**Fold change in RNA seq**	**Fold change in real-time PCR**	**Fold change in RNA seq**	**Fold change in real-time PCR**	**Fold change in RNA seq**	**Fold change in real-time PCR**
1	*RPS9*	4.27	3.23	3.72	1.87	3.97	2.71
2	*MMP12*	2.67	1.34	4.2	1.11	2.51	2.0
3	*ITGB2*	2.02	25.28	2.42	12.55	2.15	7.36
4	*CST7*	3.09	7.2	3.12	13.45	2.09	1.7
5	*SCTR*	4.26	15.78	5.18	20.11	4.13	18.25
6	*APOC1*	3.4	11.55	3.95	4.99	2.88	2.9
7	*CD69*	3.36	12.04	3.44	14.32	2.71	1.7
8	*CCNO*	2.8	5.6	3.68	11.31	3.74	3.78
9	*KERA*	−9.37	−5.55	−2.19	−7.69	−5.66	−6.6
10	*GPD1*	−6.4	−6.66	−1.62	−8.33	−6.1	−5.82
11	*EEF1A2*	−7.31	−3.3	−1.36	−4.76	−6.93	−1.5
12	*HRC*	−6.77	−4	−1.62	−6.2	−6.73	−5.8
13	*FOXD1*	−4.94	−4.34	−2.16	−8.33	−4.37	−6.25
14	*MYH1*	−10.9	−3.33	−2.36	−4.76	−10.5	−4
15	*SYNPO21*	−5.98	−5.26	−1.42	−7.1	−5.59	−3.84
16	*TNMD*	−7.12	−9	−1.88	−8.3	−7.3	−7.69
17	*MYOM2*	−7.1	−7.6	−2.61	−5.2	−6.65	−4
18	*ACTA1*	−11.5	−5.55	−1.36	−16.66	−10.1	−2.70

## Discussion

*S. aureus* and PA are known to cause biofilm-related infections and are frequently found together in chronically infected wounds, CF, CSOM, and on indwelling medical devices such as prostheses, stents, implants, catheters, and endotracheal tubes (Chole and Faddis, 2002; Hubert et al., 2013; Percival et al., 2015). Previous studies using a wound infection model suggested that poly-microbial infections of MRSA and PA were more virulent than single species infections and that synergism exists between these species, which increases virulence (Hendricks et al., 2001; Pastar et al., 2013). In various types of chronic ear infections, MRSA and PA have been frequently isolated, and these bacteria co-exist; however, their interaction with each-other or with the host was previously unknown (Klein and Chan, 2010; Kim et al., 2015). In this study, we investigated MRSA and PA multi-species biofilm communities *in vitro* and their interaction with the host during *in vivo* colonization using an OM rat model.

CV microtiter plate assay results showed significantly increased biofilm biomass in multi-species biofilms of MRSA and PA in comparison with that of single species biofilms of either MRSA or PA. It was speculated that the matrix produced by the two bacterial species and cellular components of the bacteria contributes to increased biomass. It is suggested that Staphylococcus and Pseudomonas biofilm matrix consist of extracellular DNA (e-DNA) along with exopolysaccharides (Whitchurch et al., 2002; Eckhart et al., 2007). Staphylococcal biofilm accumulation is mediated by polysaccharide intercellular adhesion (PIA), modulated by gene products encoded by *ica* operon (Cramton et al., 1999), and can also form *ica*-independent biofilm, mediated by proteins binding of the extracellular matrix (Vergara-Irigaray et al., 2009). MRSA isolates are known to produce *ica*-independent biofilms (Fitzpatrick et al., 2005). While the PA biofilms matrix consists of alginate (a negatively charged polymer of guluronic and mannuronic acid), Psl (a neutral polysaccharide consisting of a pentasaccharide repeat containing glucose, mannose, and rhamnose), and Pel (positively charged exopolysaccharide composed of partially acetylated 1 → 4 glycosidic linkages of *N*-acetylgalactosamine and *N*-acetylglucosamine) (Franklin et al., 2011). However, MRSA CFU counts were significantly lower (by >2-fold) than those of PA. These results are in agreement with previous studies that demonstrated that *S. aureus* and PA are frequently found together in human infections, and that PA kills *S. aureus* when the two species are grown together in nutrient rich medium *in vitro* (Palmer et al., 2005, 2007; Voggu et al., 2006). The killing of *S. aureus* has been attributed to various exoproducts of PA such as LasA protease (Mansito et al., 1987), HQNO (Hoffman et al., 2006), *pel* and *psl* products (Qin et al., 2009), and pyocyanin (Dietrich et al., 2006). SEM images also confirmed the presence of few MRSA bacteria in multi-species biofilms; however, increased EPS matrix was visible in multi-species biofilms, compared to that in single species biofilms. Many studies have suggested that *S. aureus* can overcome this suppression and escape PA-mediated cell death by growing as SCVs, which are characterized as a respiration-defective subpopulation with an altered phenotype (Biswas et al., 2009; Nair et al., 2014). Moreover, *S. aureus* forms SCVs in the presence of HQNO produced by PA (Hoffman et al., 2006). In addition, these SCVs that were shown to form under stress of HQNO formed robust *in vitro* biofilms through the activation of SigB and the repression of agr (Mitchell et al., 2010). Our results also showed robust biofilm formation by MRSA 3903 in the presence of HQNO, when compared to that in the control. SEM results provided further evidence of significant biofilm formation in the presence of HQNO. This process has been suggested to be due to the activation of SigB and the repression of agr, which upregulates various cell surface proteins such as FnBA, which are involved in biofilm formation (Mitchell et al., 2008, 2009, 2010).

*S. aureus* and PA frequently colonize the upper respiratory tract and cause several chronic diseases including chronic OM, cholesteatoma, chronic adenoiditis, chronic sinusitis, post-operative trampansomay, and nasal polyposis (Bendouah et al., 2006; Boase et al., 2013). Furthermore, MRSA and PA have been frequently isolated from chronic ear infections such as CSOM (Jung et al., 2009; Kim et al., 2015). In this study, we used the OM rat model to study the interaction between MRSA and PA *in vivo*. We previously used this model to study the colonization of *S. aureus* and *Streptococcus pneumoniae* separately in the rat middle ear (Yadav et al., 2012, 2014, 2015a). Herein, we first evaluated the utility of the OM rat model for multi-species colonization. The results showed consistent recovery of MRSA or PA from the rat middle ear, which was associated with significant inflammation. We choose the OM rat model over other animal models because the rat middle ear is a natural habitat for *S. aureus* and PA, and because inflammation in the middle ear mucosa (due to bacterial challenge or infection) results in severe swelling that can be easily visualized by SEM; this tissue can also be easily recovered for gene expression studies.

The middle ear is a natural habitat for many bacteria including normal body flora or opportunistic pathogens. Both *S. aureus* and PA have been isolated from middle ear infections (Brook, 2003). Our *in vivo* results showed marked recovery of MRSA or PA after multi-species and single species inoculation with significant inflammation in the rat middle ear. In addition, no significant differences in CFU counts between MRSA and PA were observed after multi-species inoculation. These results indicate that MRSA and PA successfully colonized the rat middle ear with no significant inhibition of either species. Using wound infection and rabbit models, DeLeon et al. (2014) reported no inhibition of *S. aureus* in the presence of PA during multi-species *in vivo* colonization (DeLeon et al., 2014). Other studies also showed a lack of inhibition in the absence of nutrient rich medium, which could be due to the presence of SCVs of *S. aureus* (McNamara and Proctor, 2000; Filkins et al., 2015). Inflammation in the middle ear is a sign of infection; our results also detected significant inflammation in this tissue after inoculation with either MRSA or PA alone or in combination. The significant number of bacteria recovered (based on CFU counts) indicates that multi-species bacterial colonization was established in the middle ear. SEM analysis also confirmed the presence of biofilm-like structures that were deposited on the cilia of the middle ear after single or multi-species inoculation. However, those of MRSA or PA alone were indistinguishable. Similarly, Kaya et al. (2013) also detected poly-microbial biofilms of *S. aureus* and PA in the middle ear (Saunders et al., 2011; Kaya et al., 2013). In CRS patients, multi-species biofilms have been associated with increased mucosal inflammation, more severe osteitis, higher incidence of recurrent infection (Li et al., 2011; Dong et al., 2014) and postoperative outcome (Singhal et al., 2011), and post-surgery progressions (Bendouah et al., 2006).

To analyze differential gene expression in rat middle ear mucosae inoculated with MRSA or PA alone or in combination, RNA sequencing of the total transcriptome was performed. RNA-seq results detected significantly increased expression of defense-, immune-, response-, and inflammatory-related genes after multi-species colonization and decreased expression of signaling, development, and communication pathway genes. Interestingly, a total of 122 genes (62 upregulated and 160 downregulated) were exclusively differentially regulated by multi-species colonization, and these genes were not expressed with either MRSA or PA inoculation. Gene pathway analysis revealed that those genes are involved in the phagosome, antigen processing and presentation, influenza A infection, *Staphylococcus* infection, herpes virus infection, toxoplasmosis, and tuberculosis among others.

*FCN2, CFI, KLRD1, KLRB1F, UBD, CCL5*, and *GAL* were significantly upregulated after multi-species inoculation and were either downregulated or insignificantly upregulated with MRSA or PA single inoculation. The ficolin-2-encoding gene *FCN2*, was upregulated by 1.52-fold (*P* = 0.04) with multi-species infection and was downregulated after inoculation with MRSA or PA alone. Ficolin activates the lectin pathway of complement by specifically binding to lipoteichoic acid, a cell wall constituent of gram-positive bacteria, and initiates an innate anti-microbial immune response (Lynch et al., 2004). The *CFI* gene was significantly upregulated in multi-species infections and was downregulated with MRSA or PA alone. *CFI* encodes complement factor I, which is involved in complement activation during cochlear responses to acoustic trauma (Patel et al., 2013). The *KLRD1* and *KLRB1F* genes encode the killer cell lectin-like receptor subfamily K, member 1 protein and killer cell lectin like receptor B1, which are involved in the activation of immune responses and cytotoxicity, were upregulated by 2.1- and 2.0-fold, respectively, after mixed culture inoculation, and were downregulated with MRSA or PA alone (Taniguchi et al., 2015). The *UBD* gene, encoding the protein ubiquitin D, which is involved in cytokine-induced apoptosis in rat, was upregulated by mixed cultures and MRSA and was downregulated with PA alone (Brozzi et al., 2016). Similarly, the *CCL5* gene encodes chemokine (C-C Motif) ligand 5 protein. These chemokine proteins are involved in immunoregulatory and inflammatory processes. CCL5 is an essential factor for the induction and maintenance of protective pneumococcal immunity (Palaniappan et al., 2006). The *GAL* gene encodes galanin peptides, and was upregulated by 1.9-fold or repressed with SA or PA, respectively. The galanin protein is involved in post-traumatic stress disorder and mild blast-induced traumatic brain injury (Kawa et al., 2016). The upregulation of important immune response- and cytotoxicity-related genes after multispecies inoculation indicates an enhanced host response, when compared to that after inoculation with MRSA and PA alone.

*DLECI, ST8SIA2*, and *SSC5d* are important genes, which were downregulated with multi-species inoculation and either upregulated or insignificantly downregulated with MRSA or PA alone. The *DLEC1* gene encodes the deleted in lung and esophageal cancer 1 protein (DLECI) that has tumor suppressive activities. The downregulation of this gene has been observed in several human cancers including lung, esophageal, renal, and head and neck squamous cell carcinoma (Zhang et al., 2015). Similarly, the *ST8SIA2* gene encodes alpha-2,8-sialyltransferase 2, which is involved in neuronal plasticity (Shaw et al., 2014). The *SSC5D* gene encodes scavenger receptor cysteine rich family member with 5 domains protein, which induces bacterial and fungal aggregation and subsequent inhibition of PAMP-induced cytokine release, and was also downregulated after multi-species inoculation. These results indicate that multi-species colonization triggers host responses that are different from those induced by infection with MRSA or PA alone. It has been previously reported that poly-microbial biofilms of *S. aureus* and PA, in a chronic wound infection model, significantly impair wound healing relative to that observed with their single-species biofilm counterparts. Multi-species biofilms also trigger enhanced host inflammatory responses through the expression of *IL-1*β and *TNF-*α (Seth et al., 2012). Similarly, Pastar et al. (2013) also detected significantly decreased re-epithelization with mixed species biofilms, via the suppression of keratinocyte growth factor-1. Moreover, in poly-microbial wound infections, the presence of PA induced the expression of *S. aureus* virulence factors including Panton-Valentine leucocidin and α-hemolysin (Pastar et al., 2013).

## Conclusion

This study demonstrated that MRSA and PA could co-exist in poly-microbial biofilms *in vitro*, and colonize the rat middle ear *in vivo*. The poly-microbial colonization of MRSA and PA induced the differential expression of a significant number of genes that are involved in immune response, inflammation, signaling, development, and defense; these were not expressed with single species colonization by either MSA or PA. These results indicate that poly-microbial colonization induces a host response that is different from that induced by single species infection. It is probable that poly-microbial infections were more virulent than single species infections. Thus, poly-microbial infections could modify the clinical course of disease, affecting the selection of antimicrobial therapy and the anticipated response to treatment.

## Author contributions

MY, designed research, did experiment, results analysis wrote manuscript. SC, evaluated results, chemical supply, facility arrangement, check manuscript. YG, protocol approval, animal work design, arrange reagent, and chemical facility. GI, protocol design, manuscript reading, manuscript review. JS, Protocol design, results analysis, manuscript review.

### Conflict of interest statement

The authors declare that the research was conducted in the absence of any commercial or financial relationships that could be construed as a potential conflict of interest.

## References

[B1] Anders S. Pyl P. T. Huber W. (2015). HTSeq–a python framework to work with high-throughput sequencing data. Bioinformatics 31, 166–169. 10.1093/bioinformatics/btu638 25260700 PMC4287950

[B2] Bendouah Z. Barbeau J. Hamad W. A. Desrosiers M. (2006). Biofilm formation by *Staphylococcus aureus* and *Pseudomonas aeruginosa* is associated with an unfavorable evolution after surgery for chronic sinusitis and nasal polyposis. Otolaryngol. Head Neck Surg. 134, 991–996. 10.1016/j.otohns.2006.03.001 16730544

[B3] Biswas L. Biswas R. Schlag M. Bertram R. Götz F. (2009). Small-colony variant selection as a survival strategy for *Staphylococcus aureus* in the presence of *Pseudomonas aeruginosa*. Appl. Environ. Microbiol. 75, 6910–6912. 10.1128/AEM.01211-09 19717621 PMC2772425

[B4] Boase S. Foreman A. Cleland E. Tan L. Melton-Kreft R. Pant H.. (2013). The microbiome of chronic rhinosinusitis: culture, molecular diagnostics and biofilm detection. BMC Infect. Dis. 13:210. 10.1186/1471-2334-13-210 23656607 PMC3654890

[B5] Brook I. (2003). Microbiology and management of chronic suppurative otitis media in children. J. Trop. Pediatr. 49, 196–200. 10.1093/tropej/49.4.196 12929878

[B6] Brozzi F. Gerlo S. Grieco F. A. Juusola M. Balhuizen A. Lievens S.. (2016). Ubiquitin D regulates IRE1α/c-Jun N-terminal Kinase (JNK) protein-dependent apoptosis in pancreatic beta cells. J. Biol. Chem. 291, 12040–12056. 10.1074/jbc.M115.704619 27044747 PMC4933257

[B7] Chole R. Faddis B. (2002). Evidence for microbial biofilms in cholesteatomas. Arch. Otolaryngol. Head Neck Surg. 128, 1129–1133. 10.1001/archotol.128.10.1129 12365883

[B8] Chopra S. Harjai K. Chhibber S. (2015). Antibiotic susceptibility of ica-positive and ica-negative MRSA in different phases of biofilm growth. J. Antibiot. 68, 15–22. 10.1038/ja.2014.96 25074658

[B9] Christensen G. D. Simpson W. A. Younger J. J. Baddour L. M. Barrett F. F. Melton D. M.. (1985). Adherence of coagulase-negative staphylococci to plastic tissue culture plates: a quantitative model for the adherence of staphylococci to medical devices. J. Clin. Microbiol. 22, 996–1006. 3905855 10.1128/jcm.22.6.996-1006.1985 PMC271866

[B10] Cramton S. E. Gerke C. Schnell N. F. Nichols W. W. Götz F. (1999). The intercellular adhesion (ICA) locus Is present in *Staphylococcus aureus* and is required for biofilm formation. Infect. Immun. 67, 5427–5433. 10496925 10.1128/iai.67.10.5427-5433.1999 PMC96900

[B11] DeLeon S. Clinton A. Fowler H. Everett J. Horswill A. R. Rumbaugh K. P. (2014). Synergistic interactions of *Pseudomonas aeruginosa* and *Staphylococcus aureus* in an *in vitro* wound model. Infect. Immun. 82, 4718–4728. 10.1128/IAI.02198-14 25156721 PMC4249327

[B12] Dietrich L. E. P. Price-Whelan A. Petersen A. Whiteley M. Newman D. K. (2006). The phenazine pyocyanin is a terminal signalling factor in the quorum sensing network of *Pseudomonas aeruginosa*. Mol. Microbiol. 61, 1308–1321. 10.1111/j.1365-2958.2006.05306.x 16879411

[B13] Dobin A. Davis C. A. Schlesinger F. Drenkow J. Zaleski C. Jha S.. (2013). STAR: ultrafast universal RNA-seq aligner. Bioinformatics 29, 15–21. 10.1093/bioinformatics/bts635 23104886 PMC3530905

[B14] Dong D. Yulin Z. Xiao W. Hongyan Z. Jia L. Yan X.. (2014). Correlation between bacterial biofilms and osteitis in patients with chronic rhinosinusitis. Laryngoscope 124, 1071–1077. 10.1002/lary.24424 24114791

[B15] Eckhart L. Fischer H. Barken K. B. Tolker-Nielsen T. Tschachler E. (2007). DNase1L2 suppresses biofilm formation by *Pseudomonas aeruginosa* and *Staphylococcus aureus*. Br. J. Dermatol. 156, 1342–1345. 10.1111/j.1365-2133.2007.07886.x 17459041

[B16] Fazli M. Bjarnsholt T. Kirketerp-Møller K. Jørgensen B. Andersen A. S. Krogfelt K. A.. (2009). Nonrandom distribution of *Pseudomonas aeruginosa* and *Staphylococcus aureus* in chronic wounds. J. Clin. Microbiol. 47, 4084–4089. 10.1128/JCM.01395-09 19812273 PMC2786634

[B17] Filkins L. M. Graber J. A. Olson D. G. Dolben E. L. Lynd L. R. Bhuju S.. (2015). Coculture of *Staphylococcus aureus* with *Pseudomonas aeruginosa* drives *S. aureus* towards *fermentative metabolism and reduced viability in a cystic fibrosis model*. J. Bacteriol. 197, 2252–2264. 10.1128/JB.00059-15 25917910 PMC4524177

[B18] Fitzpatrick F. Humphreys H. O'Gara J. P. (2005). Evidence for ica ADBC_independent biofilm development mechanism in methicillin-resistant *Staphylococcus aureus* clinical isolates. J. Clin. Microbiol. 43, 1973–1976. 10.1128/JCM.43.4.1973-1976.2005 15815035 PMC1081404

[B19] Flicek P. Ahmed I. Amode M. R. Barrell D. Beal K. Brent S.. (2013). Ensembl 2013. Nucleic Acids Res. 41, D48–D55. 10.1093/nar/gks1236 23203987 PMC3531136

[B20] Franklin M. J. Nivens D. E. Weadge J. T. Howell P. L. (2011). Biosynthesis of the *Pseudomonas aeruginosa* extracellular polysaccharides, alginate, Pel, and Psl. Front. Microbiol. 2:167. 10.3389/fmicb.2011.00167 21991261 PMC3159412

[B21] Gjødsbøl K. Christensen J. J. Karlsmark T. Jørgensen B. Klein B. M. Krogfelt K. A. (2006). Multiple bacterial species reside in chronic wounds: a longitudinal study. Int. Wound J. 3, 225–231. 10.1111/j.1742-481X.2006.00159.x 16984578 PMC7951738

[B22] Gutiérrez D. Ruas-Madiedo P. Martínez B. Rodríguez A. García P. (2014). Effective removal of staphylococcal biofilms by the endolysin LysH5. Rohde H. PLoS ONE 9:e107307. 10.1371/journal.pone.0107307 25203125 PMC4159335

[B23] Hall-Stoodley L. Stoodley P. (2009). Evolving concepts in biofilm infections. Cell. Microbiol. 11, 1034–1043. 10.1111/j.1462-5822.2009.01323.x 19374653

[B24] Hendricks K. J. Burd T. A. Anglen J. O. Simpson A. W. Christensen G. D. Gainor B. J. (2001). Synergy between *Staphylococcus aureus* and *Pseudomonas aeruginosa* in a rat model of complex orthopaedic wounds. J. Bone Joint Surg. 83, 855–861. 10.2106/00004623-200106000-00006 11407793

[B25] Hentzer M. Teitzel G. M. Balzer G. J. Heydorn A. Molin S. Givskov M. (2001). Alginate overproduction affects *Pseudomonas aeruginosa* biofilm structure and function. overproduction affects *Pseudomonas aeruginosa* biofilm structure and function. *J*. Bacteriol. 183, 5395–401. 10.1128/JB.183.18.5395-5401.2001 PMC95424 11514525

[B26] Hoffman L. R. Deziel E. D'Argenio D. A. Lepine F. Emerson J. McNamara S.. (2006). Selection for *Staphylococcus aureus* small-colony variants due to growth in the presence of *Pseudomonas aeruginosa*. Proc. Natl. Acad. Sci. U.S.A. 103, 19890–19895. 10.1073/pnas.0606756104 17172450 PMC1750898

[B27] Hubert D. Réglier-Poupet H. Sermet-Gaudelus I. Ferroni A. Le Bourgeois M. Burgel P.-R.. (2013). Association between *Staphylococcus aureus* alone or combined with *Pseudomonas aeruginosa* and the clinical condition of patients with cystic fibrosis. J. Cyst. Fibros. 12, 497–503. 10.1016/j.jcf.2012.12.003 23291443

[B28] Jung H. Lee S. K. Cha S.-H. Byun J. Y. Park M. S. Yeo S. G. (2009). Current bacteriology of chronic otitis media with effusion: high rate of nosocomial infection and decreased antibiotic sensitivity. J. Infect. 59, 308–316. 10.1016/j.jinf.2009.08.013 19715725

[B29] Kawa L. Barde S. Arborelius U. P. Theodorsson E. Agoston D. Risling M.. (2016). Expression of galanin and its receptors are perturbed in a rodent model of mild, blast-induced traumatic brain injury. Exp. Neurol. 279, 159–167. 10.1016/j.expneurol.2016.02.019 26928087

[B30] Kaya E. Dag I. Incesulu A. Gurbuz M. K. Acar M. Birdane L. (2013). Investigation of the presence of biofilms in chronic suppurative otitis media, nonsuppurative otitis media, and chronic otitis media with cholesteatoma by scanning electron microscopy. Sci. World J. 2013:638715. 10.1155/2013/638715 24288500 PMC3826458

[B31] Kim S. H. Kim M. G. Kim S. S. Cha S. H. Yeo S. G. (2015). Change in detection rate of methicillin-resistant *Staphylococcus aureus* and *Pseudomonas aeruginosa* and their antibiotic sensitivities in patients with chronic suppurative otitis media. J. Int. Adv. Otol. 11, 151–156. 10.5152/iao.2015.1106 26381007

[B32] Klein J. Chan S. (2010). Methicillin-resistant *Staphylococcus aureus* in middle ear fluid of children. Clin. Pediatr. 49, 66–68. 10.1177/0009922809342463 19628757

[B33] Li H. Wang D. Sun X. Hu L. Yu H. Wang J. (2011). Relationship between bacterial biofilm and clinical features of patients with chronic rhinosinusitis. Eur. Arch. Oto Rhino Laryngol. 269, 155–163. 10.1007/s00405-011-1683-y 21739098

[B34] Li J. Turnidge J. Milne R. Nation R. L. Coulthard K. (2001). *In vitro* pharmacodynamic properties of colistin and colistin methanesulfonate against *Pseudomonas aeruginosa* isolates from patients with cystic fibrosis. Antimicrob. Agents Chem. 45, 781–785. 10.1128/AAC.45.3.781-785.2001 11181360 PMC90373

[B35] Lister J. L. Horswill A. R. (2014). *Staphylococcus aureus* biofilms: recent developments in biofilm dispersal. Front. Cell. Infect. Microbiol. 4:178. 10.3389/fcimb.2014.00178 25566513 PMC4275032

[B36] Livak K. J. Schmittgen T. D. (2001). Analysis of relative gene expression data using real-time quantitative PCR and the 2^−ΔΔCT^ Method. Methods 25, 402–408. 10.1006/meth.2001.1262 11846609

[B37] Lyczak J. B. Cannon C. L. Pier G. B. (2002). Lung infections associated with cystic fibrosis. Clin. Microbiol. Rev. 15, 194–222. 10.1128/CMR.15.2.194-222.2002 11932230 PMC118069

[B38] Lynch N. J. Roscher S. Hartung T. Morath S. Matsushita M. Maennel D. N.. (2004). L-Ficolin specifically binds to lipoteichoic acid, a cell wall constituent of gram-positive bacteria, and activates the lectin pathway of complement. J. Immunol. 172, 1198–1202. 10.4049/jimmunol.172.2.1198 14707097

[B39] Mansito T. B. Falcón M. A. Moreno J. Carnicero A. Gutierrez-Navarro A. M. (1987). Effects of staphylolytic enzymes from *Pseudomonas aeruginosa* on the growth and ultrastructure of *Staphylococcus aureus*. Microbios 49, 55–64. 3104732

[B40] McNamara P. J. Proctor R. A. (2000). *Staphylococcus aureus* small colony variants, electron transport and persistent infections. Int. J. Antimicrob. Agents 14, 117–122. 10.1016/S0924-8579(99)00170-3 10720801

[B41] Mitchell G. Brouillette E. Seguin D. L. Asselin A. E. Jacob C. L. Malouin F. (2009). A role for sigma factor B in the emergence of Staphylococcus aureus small-colony variants and elevated biofilm production resulting from an exposure to aminoglycosides. Microb. Pathog. 48, 18–27. 10.1016/j.micpath.2009.10.003 19825410

[B42] Mitchell G. Lamontagne C. A. Brouillette E. Grondin G. Talbot B. G. Grandbois M.. (2008). Staphylococcus aureus SigB activity promotes a strong fibronectin-bacterium interaction which may sustain host tissue colonization by small-colony variants isolated from cystic fibrosis patients. Mol. Microbiol. 70, 1540–1555. 10.1111/j.1365-2958.2008.06511.x 19007412

[B43] Mitchell G. Séguin D. L. Asselin A.-E. Déziel E. Cantin A. M. Frost E. H.. (2010). *Staphylococcus aureus* sigma B-dependent emergence of small-colony variants and biofilm production following exposure to *Pseudomonas aeruginosa* 4-hydroxy-2-heptylquinoline-N- oxide. BMC Microbiol. 10:33. 10.1186/1471-2180-10-33 20113519 PMC2824698

[B44] Nair N. Biswas R. Götz F. Biswas L. (2014). Impact of *Staphylococcus aureus* on pathogenesis in polymicrobial infections. Infect. Immun. 82, 2162–2169. 10.1128/IAI.00059-14 24643542 PMC4019155

[B45] Palaniappan R. Singh S. Singh U. P. Singh R. Ades E. W. Briles D. E.. (2006). CCL5 Modulates pneumococcal immunity and carriage. J. Immunol. 176, 2346–2356. 10.4049/jimmunol.176.4.2346 16455992

[B46] Palmer K. L. Aye L. M. Whiteley M. (2007). Nutritional cues control *Pseudomonas aeruginosa* multicellular behavior in cystic fibrosis sputum. J. Bacteriol. 189, 8079–8087. 10.1128/JB.01138-07 17873029 PMC2168676

[B47] Palmer K. L. Mashburn L. M. Singh P. K. Whiteley M. (2005). Cystic fibrosis sputum supports growth and cues key aspects of *Pseudomonas aeruginosa* physiology. J. Bacteriol. 187, 5267–5277. 10.1128/JB.187.15.5267-5277.2005 16030221 PMC1196007

[B48] Pastar I. Nusbaum A. G. Gil J. Patel S. B. Chen J. Valdes J.. (2013). Interactions of methicillin resistant *Staphylococcus aureus* USA300 and *Pseudomonas aeruginosa* in polymicrobial wound infection. PLoS ONE 8:e56846. 10.1371/journal.pone.0056846 23451098 PMC3579943

[B49] Patel M. Hu Z. Bard J. Jamison J. Cai Q. Hu B. H. (2013). Transcriptome characterization by RNA-Seq reveals the involvement of the complement components in noise-traumatized rat cochleae. Neuroscience 248, 1–16. 10.1016/j.neuroscience.2013.05.038 23727008 PMC3868636

[B50] Percival S. L. Suleman L. Vuotto C. Donelli G. (2015). Healthcare-associated infections, medical devices and biofilms: risk, tolerance and control. J. Med. Microbiol. 64, 323–334. 10.1099/jmm.0.000032 25670813

[B51] Post J. C. Stoodley P. Hall–Stoodley L. Ehrlich G. D. (2004). The role of biofilms in otolaryngologic infections. Curr. Opin. Otolaryngol. Head Neck Surg. 12, 185–190. 10.1097/01.moo.0000124936.46948.6a 15167027

[B52] Qin Z. Yang L. Qu D. Molin S. Tolker-Nielsen T. (2009). *Pseudomonas aeruginosa* extracellular products inhibit staphylococcal growth, and disrupt established biofilms produced by *Staphylococcus epidermidis*. Microbiology 155, 2148–2156. 10.1099/mic.0.028001-0 19389780

[B53] Saunders J. Murray M. Alleman A. (2011). Biofilms in chronic suppurative otitis media and cholesteatoma: scanning electron microscopy findings. Am. J. Otolaryngol. 32, 32–37. 10.1016/j.amjoto.2009.09.010 20036033

[B54] Seth A. K. Geringer M. R. Hong S. J. Leung K. P. Galiano R. D. Mustoe T. A. (2012). Comparative analysis of single-species and polybacterial wound biofilms using a quantitative, *in vivo* rabbit ear model. PLoS ONE 7:e42897. 10.1371/journal.pone.0042897 22905182 PMC3414496

[B55] Shaw A. D. Tiwari Y. Kaplan W. Heath A. Mitchell P. B. Schofield P. R.. (2014). Characterisation of genetic variation in ST8SIA2 and its interaction region in NCAM1 in patients with bipolar disorder. PLoS ONE 9:e92556. 10.1371/journal.pone.0092556 24651862 PMC3961385

[B56] Singhal D. Foreman A. Bardy J.-J. Wormald P.-J. (2011). *Staphylococcus aureus* biofilms. Laryngoscope 121, 1578–1583. 10.1002/lary.21805 21647904

[B57] Sun J. Nishiyama T. Shimizu K. Kadota K. (2013). TCC: an R package for comparing tag count data with robust normalization strategies. BMC Bioinformatics 14:219. 10.1186/1471-2105-14-219 23837715 PMC3716788

[B58] Taniguchi R. Koyano S. Suzutani T. Goishi K. Ito Y. Morioka I.. (2015). A Thr72Ala polymorphism in the NKG2D gene is associated with early symptomatic congenital cytomegalovirus disease. Infection 43, 353–359. 10.1007/s15010-015-0774-x 25861030

[B59] Trapnell C. Williams B. A. Pertea G. Mortazavi A. Kwan G. van Baren M. J.. (2010). Transcript assembly and abundance estimation from RNA-Seq reveals thousands of new transcripts and switching among isoforms. Nat. Biotechnol. 28, 511–515. 10.1038/nbt.1621 20436464 PMC3146043

[B60] Vergara-Irigaray M. Valle J. Merino N. Latasa C. García B. Ruiz-de L. M.. (2009). Relevant role of fibronectin-binding proteins in *Staphylococcus aureus* biofilm-associated foreign-body infections. Infect. Immun. 77, 3978–3991. 10.1128/IAI.00616-09 19581398 PMC2738049

[B61] Voggu L. Schlag S. Biswas R. Rosenstein R. Rausch C. Götz F. (2006). Microevolution of cytochrome bd oxidase in staphylococci and its implication in resistance to respiratory toxins released by pseudomonas. J. Bacteriol. 188, 8079–8086. 10.1128/JB.00858-06 17108291 PMC1698191

[B62] Whitchurch C. B. Tolker-Nielsen T. Ragas P. C. Mattick J. S. (2002). Extracellular DNA required for bacterial biofilm formation. Science 295:1487. 10.1126/science.295.5559.1487 11859186

[B63] Wozniak D. J. Wyckoff T. J. Starkey M. Keyser R. Azadi P. O'Toole G. A.. (2003). Alginate is not a significant component of the extracellular polysaccharide matrix of PA14 and PAO1. *Pseudomonas aeruginosa* biofilms. Proc. Natl. Acad. Sci. U.S.A. 100, 7907–7912. 10.1073/pnas.1231792100 12810959 PMC164686

[B64] Yadav M. K. Chae S.-W. Im G. J. Chung J.-W. Song J.-J. (2015a). Eugenol: a phyto-compound effective against methicillin-resistant and methicillin-sensitive *Staphylococcus aureus* clinical strain biofilms. PLoS ONE 10:e0119564. 10.1371/journal.pone.0119564 25781975 PMC4364371

[B65] Yadav M. K. Chae S.-W. Song J.-J. (2012). *In Vitro Streptococcus pneumoniae* biofilm formation and *in vivo* middle ear mucosal biofilm in a rat model of acute otitis induced by S. pneumoniae. Clin. Exp. Otorhinolaryngol. 5, 139–144. 10.3342/ceo.2012.5.3.139 22977710 PMC3437414

[B66] Yadav M. K. Go Y. Y. Chae S.-W. Song J.-J. (2015b). The small molecule dam inhibitor, pyrimidinedione, disrupts *Streptococcus pneumoniae* biofilm growth *in vitro*. PLoS ONE 10:e0139238. 10.1371/journal.pone.0139238 26431532 PMC4592238

[B67] Yadav M. K. Park S.-W. Chae S.-W. Song J.-J. (2014). Sinefungin, a natural nucleoside analogue of s-adenosylmethionine, inhibits *Streptococcus pneumoniae* biofilm growth. Biomed Res. Int. 2014:156987. 10.1155/2014/156987 25050323 PMC4094849

[B68] Zhang L. Zhang Q. Li L. Wang Z. Ying J. Fan Y.. (2015). DLEC1, a 3p tumor suppressor, represses NF-κB signaling and is methylated in prostate cancer. J. Mol. Med. 93, 691–701. 10.1007/s00109-015-1255-5 25648635

